# Meta-Analysis and Physiologically-Based Modeling of the Pharmacokinetics and Pharmacodynamics of Dexamethasone in Horses

**DOI:** 10.1007/s11095-026-04120-5

**Published:** 2026-06-19

**Authors:** Ruihong Yu, Pierre-Louis Toutain, Carl Ekstrand, William J. Jusko

**Affiliations:** 1 Division of Pharmacokinetics, Pharmacodynamics, and Systems Pharmacology, Department of Pharmaceutical Sciences, School of Pharmacy and Pharmaceutical Sciences, State University of New York at Buffalo, Buffalo, NY USA 404 Pharmacy Building, 14214-8033; 2 Department of Comparative Biomedical Sciences, The Royal Veterinary College, University of London, London, UK; 3 Department of Animal Biosciences, Swedish University of Agricultural Sciences, Uppsala, Sweden

**Keywords:** adrenal suppression, dexamethasone, glucose, pharmacodynamics, pharmacokinetics, physiologically based pharmacokinetic (PBPK) model

## Abstract

**Purpose:**

Dexamethasone (DEX) is widely used in equine practice for its potent anti-inflammatory effects and diverse studies have examined its pharmacology in horses. We integrated all available pharmacokinetic (PK) and pharmacodynamic (PD) data from 12 studies to quantify DEX disposition and endocrine effects in horses.

**Methods:**

DEX concentrations in blood, urine and synovial fluid, plus cortisol (CTS) and glucose (GLU) in plasma, following various administration routes (intravenous (IV), intramuscular (IM), intra-articular, oral) were available from original studies or digitized from literature. A minimal physiologically-based PK model and linked indirect response PD models were applied.

**Results:**

The mean clearance of DEX was 344 mL/h/kg via hepatic metabolism (98%) and renal excretion (2%). Due to nonlinear tissue binding, DEX generally exhibited a prolonged terminal phase in plasma, maintaining concentrations above a designated plasma threshold of 5 pg/mL for 67 h following 0.05 mg/kg IV dose. Dosing input parameters of DEX varied markedly across dosing routes and prodrug formulations (alcohol, isonicotinate, phosphate), with bioavailability ranging 37 ~ 100%. Oral and pro-drug doses produced rapid absorption, except for IM DEX-isonicotinate that exhibited slow (flip-flop) availability. Adrenal suppression with an *IC*_*50*_ of 0.038 ng/mL and plasma GLU increases with an *EC*_*50*_ of 0.79 ng/mL were observed that commonly persisted for 2 ~ 4 days after single dose.

**Conclusions:**

This meta-analysis utilized a mechanistic and physiologically-based modeling framework to provide global perspectives that may promote the rational use of DEX in equine medicine and support evidence-based regulatory decisions.

**Graphical Abstract:**

**Figure:**
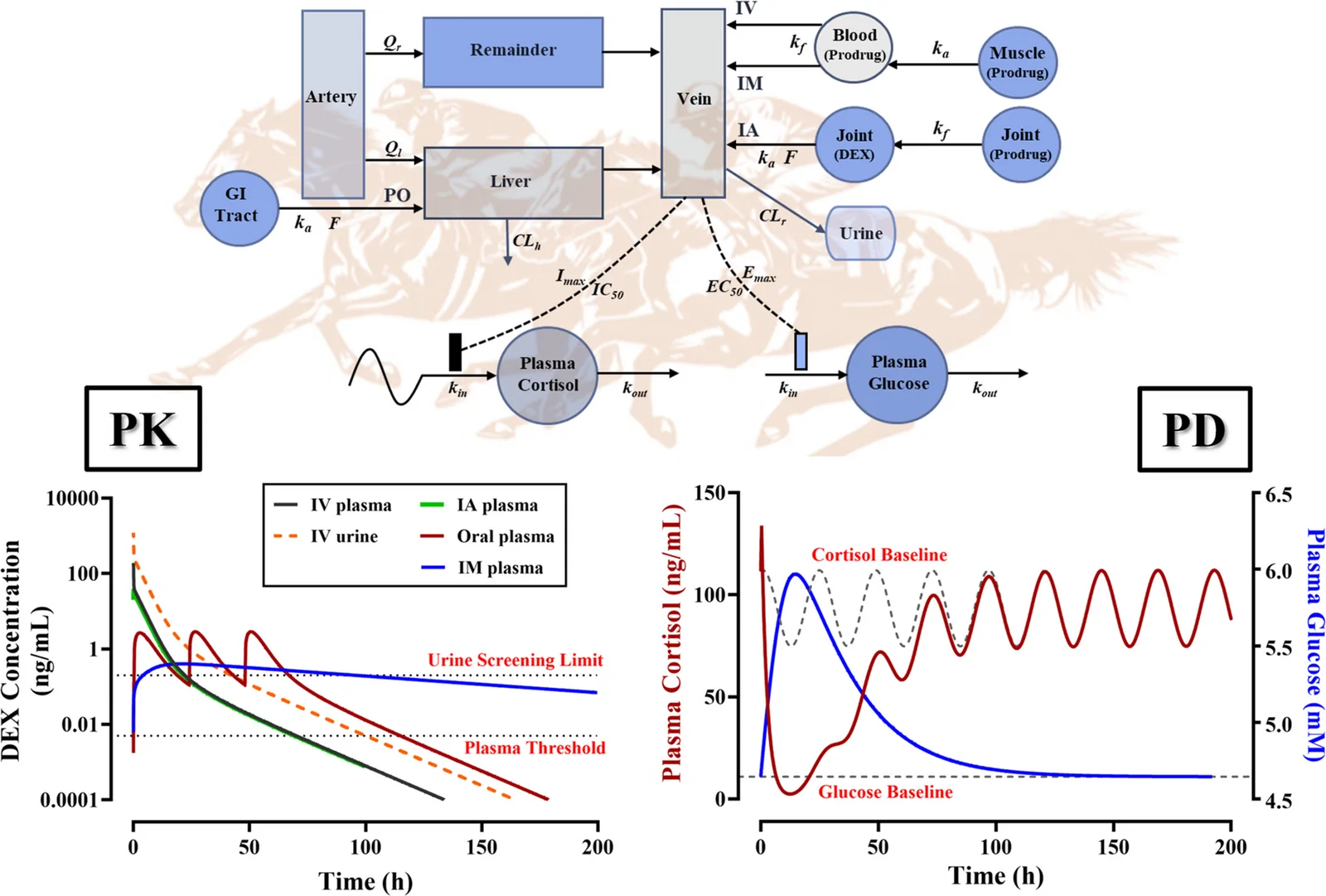

**Supplementary Information:**

The online version contains supplementary material available at https://doi.org/10.1007/s11095-026-04120-5.

## Introduction

Dexamethasone (DEX) is a synthetic glucocorticoid (GC) commonly used in equine veterinary medicine to manage a variety of clinical conditions, including pulmonary diseases (e.g. equine asthma, interstitial pneumonia), local musculoskeletal inflammations (e.g. osteoarthritis, synovitis, tendonitis), hypersensitivity reactions, uveitis, and others. It acts by specifically binding to glucocorticoid receptors (GR), subsequently inhibiting the production of proinflammatory cytokines and thereby exerting potent anti-inflammatory and immunosuppressive effects. Compared to endogenous cortisol (CTS, also known as hydrocortisone), DEX is 25 ~ 30 times more potent [1, 2]. It is available as both alcohol and ester forms, such as DEX sodium phosphate (DEX_PHO), DEX isonicotinate (DEX_ISO), and DEX acetate, and has been given to horses via various routes, including intravenous (IV), oral (PO), intra-articular (IA), intramuscular (IM), dermal (as an ointment) [3], topical [4] and nebulized formulations [5]. The recommended equine dosages are 0.05 ~ 0.06 mg/kg for Dexadreson® (DEX_PHO; Intervet UK Ltd) and 0.02 mg/kg for IM Voren® suspension (DEX_ISO; Boehringer Ingelheim Ltd).

DEX use in performance and racehorses is commonplace, albeit with certain concerns. Distinguishing its legitimate use for medical treatment and illegal use as a doping agent can be challenging [6, 7]. While DEX is effective in reducing inflammatory symptoms, it does not address the underlying causes of disease and may mask existing injuries or pathologies [8]. To safeguard animal welfare and uphold the integrity of the horse racing sport, DEX use in horses is tightly regulated. For instance, many North American racing authorities have adopted a regulatory threshold of 5 pg/mL in plasma or serum above which horses should not race [9, 10].

The pharmacokinetics (PK) of DEX has been studied for various formulations and dosing routes in healthy horses. However, its anti-inflammatory effects and pharmacodynamics (PD) have limited studies. As an alternative, some adverse effects following DEX use have been monitored and utilized as surrogate markers for PD evaluation. The most commonly used index is adrenal suppression, followed by hyperglycemia [11, 12], elevated lactate [13], and thyroid suppression [14]. Exogenous GC dosing suppresses the hypothalamic–pituitary–adrenal (HPA) axis reducing the synthesis and secretion of cortisol [15]. Cortisol secretion may also be directly inhibited by DEX at the adrenal cortex, independent of the HPA axis [16]. The potential mechanism by which DEX induces hyperglycemia relates to stress hormones playing a key role in maintaining glucose (GLU) homeostasis [17]. By stimulating hepatic gluconeogenesis and inhibiting glucose uptake and utilization in muscle and adipose tissue, GC helps preserve optimal brain function during periods of stress.

By reviewing and synthesizing all available published data on DEX and associated biomarkers and incorporating perspectives from the literature, we developed minimal physiologically-based PK (mPBPK) and linked PD models to quantitatively describe and predict DEX disposition and effects in horses. These modeling and simulation-based approaches may support evidence-based optimization of DEX dosing regimens in equine clinical settings and may also offer useful insights for refining anti-doping and medication control regulations.

## Methods

### Data Collection and Processing

Relevant literature search for DEX PK and PD in horses was performed in PubMed using the keywords ‘‘((pharmacokinetics) OR (plasma) OR (concentration)) AND (dexamethasone) AND (horse)’’ and “((cortisol) OR (hydrocortisone) OR (glucose) OR (pharmacodynamics)) AND (dexamethasone) AND (horse)’’, last accessed June 2025, plus reference tracing. The DEX concentrations in plasma, synovial fluid (SF), and urine, as well as CTS and GLU concentrations in plasma, were digitized from published graphs using Gsys2.4 (https://www.jcprg.org/gsys/) or directly obtainable for these studies as previously published by the authors.

### DEX mPBPK Model Development

**Fig. 1:**
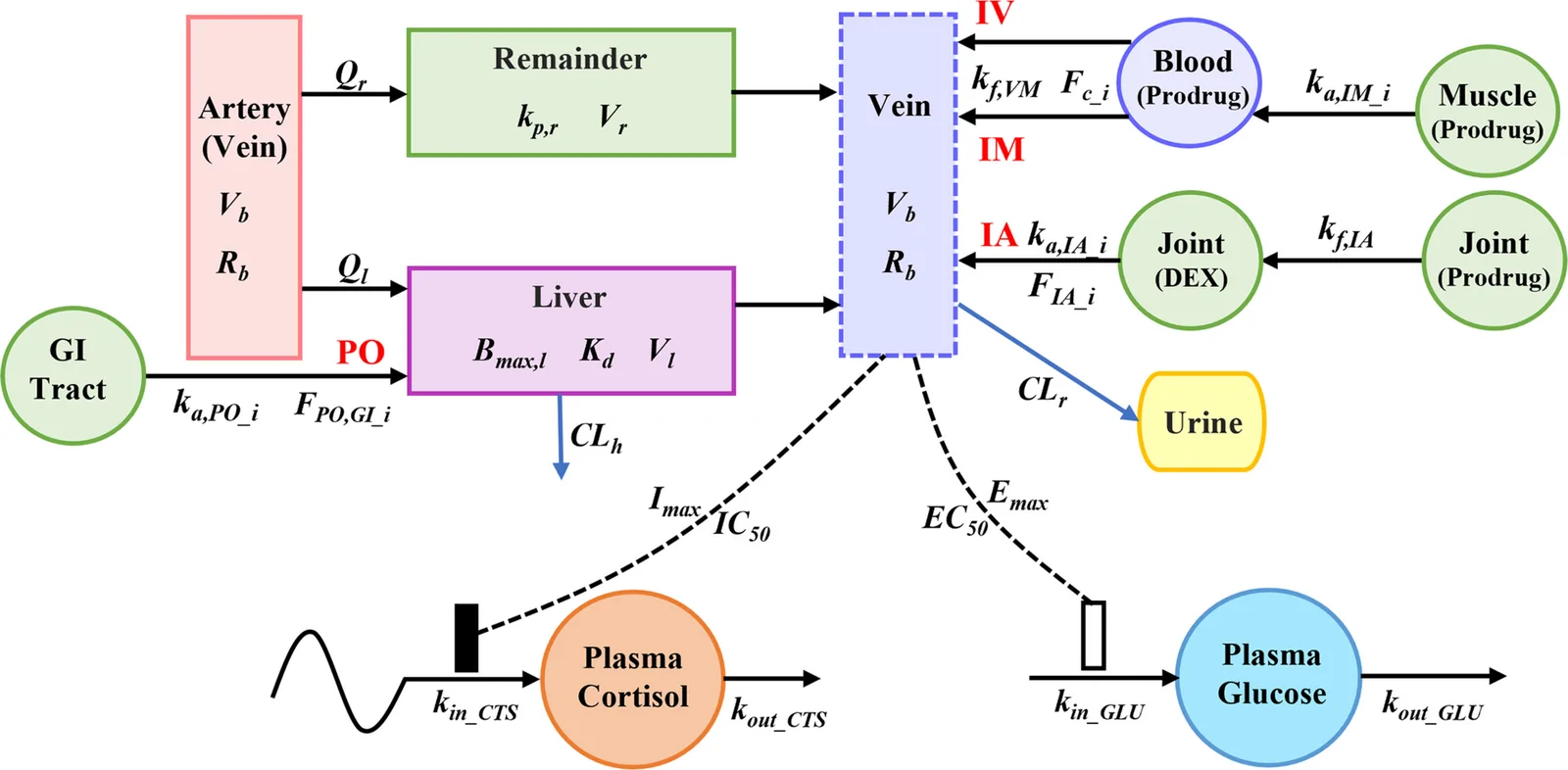
Minimal physiologically based pharmacokinetic and pharmacodynamic model of dexamethasone (DEX) following intravenous (IV), intramuscular (IM), intra-articular (IA), and intragastric (PO) dosing

The schematic of the proposed mPBPK model structure for DEX in horses is shown in the upper section of Fig. 1, which is based on our methylprednisolone (MPL) PK models [18, 19]. Briefly, it consists of vein, artery, liver, remainder tissue, and urine compartments, incorporating nonlinear tissue binding in the liver, with clearance occurring through hepatic metabolism and renal excretion to account for the dual elimination pathways. The input processes varied by administration routes: prodrug conversion was assumed in blood for IV and IM doses due to lack of carboxylesterase in muscle, and was assumed incomplete due to systemic prodrug loss, while prodrugs underwent complete conversion into DEX within joints after IA injection. Absorption of prodrugs from the IM depot and generated DEX from IA joints was described by first-order kinetics. Oral absorption was linked to the liver and mechanistically quantify the hepatic first-pass effect.

The prodrug (*i* = DEX_PHO and DEX_ISO) changes in blood following IV dosing was:

1 $$\begin{array}{cc}\frac{dA_{blood\_i}}{dt}={-k}_{f,VM\_i}\cdot A_{blood\_i}\quad\quad\quad{A}_{blood\_i}\left(0\right)={dose}_i\end{array}$$

For IM doses, the prodrug (*i* = DEX_PHO and DEX_ISO) changes in the dosing depot and blood were:

2 $$\begin{array}{cc}\frac{d{A}_{depot\_i}}{dt}={- k}_{a,IM\_i}\cdot {A}_{depot\_i}\quad\quad\quad{A}_{depot\_i}\left(0\right)={dose}_{i}\end{array}$$

3 $$\begin{array}{cc}\frac{d{A}_{blood\_i}}{dt}={ k}_{a,IM\_i}\cdot {A}_{depot\_i}-{k}_{f,VM\_i}\cdot {A}_{blood\_i}\quad\quad\quad {A}_{blood\_i}\left(0\right)=0\end{array}$$

DEX amount in the dosing depot after oral doses (*i* = tablet and solution) was:

4 $$\begin{array}{cc}\frac{d{A}_{depot\_DEX}}{dt}={-k}_{a,PO\_i}\cdot {A}_{depot\_DEX}\quad\quad\quad{A}_{depot\_DEX}\left(0\right)={dose}_{DEX}\end{array}$$

For IA doses, the time course of prodrug (*i* = DEX_PHO) amount and DEX concentration in the synovial fluid (SF) of healthy (H) and inflamed (I) joints were:

5 $$\begin{array}{cc}\frac{d{A}_{i,SF\_H}}{dt}={- k}_{f,IA\_i}\cdot {A}_{i,SF\_H}\quad\quad\quad{A}_{H,SF\_i}\left(0\right)={dose}_{i}\end{array}$$

6 $$\begin{aligned}\frac{dA_{DEX,SF\_H}}{dt}=&{k_{f,IA\_i}\cdot A}_{i,SF\_H}\cdot\frac{{MW}_{DEX}}{{MW}_i}\\&{-k}_{a,IA\_DEX}\cdot A_{DEX,SF\_H}\\&A_{DEX,SF\_H}\left(0\right)=0\end{aligned}$$

7 $${A}_{DEX, SF\_I}={dose}_{DEX}\cdot \left({f}_{1}\cdot {e}^{-\alpha \cdot t}+ {f}_{2}\cdot {e}^{-\beta \cdot t}+ {f}_{3}\cdot {e}^{-\gamma \cdot t}\right)$$

where *k*_*f*_ is the formation rate constant of DEX from the prodrugs for the indicated dosing routes, and *k*_*a*_ is the absorption rate constant. The *f*_*1*_, *f*_*2*_, and *f*_*3*_ are the fractional intercepts for the three exponential phases with corresponding slopes *α*, *β*, and *γ*. The *V*_*SF*_ is the synovial fluid volume for the horse radiocarpal joint plus drug solution volume, 16.9 mL [20].

The differential equations for DEX concentrations in blood (*C*_*p*_), liver (*C*_*l*_), and remainder tissues (*C*_*r*_) are:

8 $$\begin{aligned}\frac{d{C}_{p}}{dt}\cdot {V}_{b}\cdot {R}_{b}&={input}_{IV/IM/IA}+{Q}_{l}\cdot \frac{{C}_{l}}{{f}_{ut}}+{Q}_{r}\cdot \frac{{C}_{r}}{{K}_{p,r}}\\&-{Q}_{co}\cdot {C}_{p}\cdot {R}_{b}{{-CL}_{r}\cdot C}_{p};\,\,\,{ C}_{p}\left(0\right)=0\\ {input}_{IV/IM}&= {{F}_{VM\_i}\cdot k}_{f,VM\_i}\cdot {A}_{blood\_i}\cdot \frac{{MW}_{DEX}}{{MW}_{i}}\\ {input}_{IA}&= {{F}_{IA\_i}\cdot k}_{a,IA\_DEX}\cdot {A}_{DEX, SF}\end{aligned}$$

9 $$\begin{aligned}\frac{d{C}_{l}}{dt}\cdot {V}_{l}=&{{input}_{PO}+Q}_{l}\cdot \left({C}_{p}\cdot {R}_{b}-\frac{{C}_{l}}{{f}_{ut}}\right)-{CL}_{h,int}\cdot \frac{{C}_{l}}{{K}_{pu,l}}\\&{C}_{l}\left(0\right)=\\&0{input}_{PO}= {{F}_{GI}\cdot k}_{a,PO\_i}\cdot {A}_{depot\_DEX}\end{aligned}$$

10 $$\begin{array}{cc}\frac{d{C}_{r}}{dt}\cdot {V}_{r}={Q}_{r}\cdot \left({C}_{p}\cdot {R}_{b}-\frac{{C}_{r}}{{K}_{p,r}}\right)\quad\quad\quad {C}_{r}\left(0\right)=0\end{array}$$

where *F* is bioavailability due to incomplete prodrug conversion or DEX absorption, *F*_*GI*_ specifically indicates gastrointestinal (GI) tract bioavailability; *V*_*b*_, *V*_*l*_, and *V*_*r*_ are volumes of the indicated compartments; *Q*_*co*_, *Q*_*l*_, and *Q*_*r*_ are cardiac output, total blood flow to liver, and total blood flow to the remainder tissues. As is typical in most PBPK models, the measured venous drug concentration was assumed to reflect the arterial drug concentration. Doses were adjusted for the molecular weights (MW) of DEX (392.46), DEX_PHO (516.41), and DEX_ISO (492.54). The values of the above physiologic parameters in horses are provided in Table S1 of the Supplementary Materials [18]. The blood-to-plasma ratio (*R*_*b*_) of DEX in horses is 0.69 from a rat study [21]. The *CL*_*r*_ and *CL*_*h,int*_ are the renal clearance and intrinsic hepatic clearance, and *K*_*p,r*_ is the unbound or total tissue/plasma partition coefficients for liver and remainder tissues. Liver binding (*f*_*ut*_) was depicted as [22]:

11 $$\frac{1}{{f}_{ut}}= \frac{\sqrt{{\left({C}_{l}-{K}_{d}- {B}_{max,l}\right)}^{2}+4\cdot { K}_{d}{\cdot C}_{l}}-\left({C}_{l}-{K}_{d}- {B}_{max,l}\right)}{2\cdot { K}_{d}}$$

in which *B*_*max,l*_, *K*_*d*_, and* C*_*l*_ represent binding capacity, equilibrium dissociation constant, and total concentration of DEX in the liver.

The urine concentrations of DEX (*C*_*u*_) at midpoints of collection intervals were expressed as:

12 $$\frac{{C}_{u,i}+{C}_{u,i+1}}{2} ={{CL}_{r}\cdot C}_{p}/{V}_{u}$$

where *V*_*u*_ is the averaged urine flow of horse per hour, 494 mL/h [23].

### PD Model for Adrenal Suppression

The PD model used to characterize plasma CTS concentrations is presented in Fig. 1. Endogenous plasma CTS exhibits a symmetrical circadian pattern [24, 25] and can be depicted as a cosine function:

13 $$R_{bl\_CTS}\left(t\right)=R_m+R_a\cdot cos\left(\frac{2\mathrm\pi}T\cdot\left(t-t_p\right)\right)$$

where *R*_*m*_ and *R*_*a*_ are the mean baseline and amplitude, *T* is the biorhythmic period (24 h), and *t*_*p*_ is the peak time or acrophase. In the absence of DEX, plasma CTS is governed by a time-varying synthesis, *k*_*in_CTS*_(t), and first-order dissipation, *k*_*out_CTS*_:

14 $$\frac{d{R}_{bl\_CTS}}{dt}={k}_{in\_CTS}\left(t\right)-{k}_{out\_CTS}\cdot {R}_{bl\_CTS}\left(t\right)$$

Combining Eqs. 13 and 14 yields:

15 $$\begin{aligned}k_{in\_CTS}\left(t\right)&=k_{out\_CTS}\cdot R_m+k_{out\_CTS}\cdot R_a\cdot cos\left(\frac{2\mathrm\pi}T\cdot\left(t-t_p\right)\right)\\&-\frac{2\mathrm\pi}T\cdot R_a\cdot sin\left(\frac{2\mathrm\pi}T\cdot\left(t-t_p\right)\right)\end{aligned}$$

The plasma concentration of DEX (*C*_*p*_) was used as the driving force for adrenal suppression. Additionally, external disturbances such as animal handling, restraint, and injection pain can stimulate a transient CTS impulse. Thus, the mechanism-based effects of DEX on CTS were depicted using indirect response model (IDR) I, which assumes inhibition of the production process [26]:

16 $$\begin{array}{c}\frac{d{R}_{DEX\_CTS}}{dt}=\frac{{R}_{max, stress\_i}}{\tau }+{k}_{in\_CTS}\left(t\right)\cdot \left(1-\frac{{{I}_{max}\cdot C}_{p}}{{C}_{ p}+{IC}_{50}}\right)-{k}_{out\_CTS}\cdot {R}_{DEX\_CTS}\\ \begin{array}{cc}\frac{{R}_{max, stress\_1}}{\tau }=0& when \,t\end{array}\ge \tau \end{array}$$

where *IC*_*50*_ is the plasma concentration of DEX causing 50% inhibition of CTS production, *I*_*max*_ is its maximal inhibition, and *R*_*max,stress*_ is the maximal stress-induced response, which operates for the duration of *τ* after drug dosing.

### PD Model for Plasma Glucose

The PD model used to characterize plasma glucose concentrations after DEX administration is shown in Fig. 1. Glucocorticoids exert their plasma GLU response primarily by increasing the rate of gluconeogenesis [27], and therefore the time course of DEX-induced plasma GLU change was depicted using IDR III, which assumes stimulation of the production process [26]:

17 $$\frac{d{R}_{DEX\_GLU}}{dt}={k}_{in\_GLU}\cdot \left(1+\frac{{E}_{max }\cdot {C}_{p}}{{C}_{p}+{EC}_{50}}\right)-{k}_{out\_GLU}\cdot {R}_{DEX\_GLU}$$

in which *E*_*max*_ and *EC*_*50*_ are the maximal stimulation of DEX induced GLU production and the corresponding plasma concentration that produces 50% of this maximal effect, *k*_*in_GLU*_ and *k*_*out_CTS*_ are the zero-order production rate and first-order loss rate constant of GLU. The initial condition of the equation is the baseline value at time zero.

### Data Analysis

A six-step sequential PK/PD modeling strategy was implemented. 1) The mean plasma and urine concentration–time data of DEX from the various studies in healthy horses was simultaneously fitted with the proposed mPBPK model, as described by Eqs. 1~6 and 8~10. 2) A separate mPBPK model fitting based on Eqs. 7~11 was performed for the SF and plasma DEX data from the LPS study of IA doses in inflamed joints. Here, only absorption-related parameters were estimated, while others were fixed. 3) PK simulations were conducted for the doses with sparse, previously unused urine data (2 ~ 3 timepoints per profile), serving as a PK model qualification step. The PK parameters obtained from the mPBPK modeling were then fixed for the remaining PD analysis. 4) All mean plasma CTS concentration–time data from healthy animals was fitted using Eqs. 14 ~ 16, including baseline and drug effect. 5) CTS profiles following IA dosing in horses with experimentally-induced synovitis were simulated based on mPBPK/PD parameters obtained in prior steps. Given that these data were derived from single animals and exhibited high variability, this simulation was used to support PD model qualification. 6) Finally, the entire array of GLU data was jointly analyzed using Eq. 17.

Overall model fittings were implemented using the maximum likelihood method in ADAPT 5 [28]. The variance model was:

18 $${V}_{i}={({\sigma}_{1}+{\sigma}_{2}\cdot {Y}_{i})}^{2}$$

where *V*_*i*_ represents the variance of the *i*th data point, *Y*_*i*_ is the *i*th model prediction, and *σ*_*1*_ as well as *σ*_*2*_ are variance model parameters. The performance of model was evaluated by goodness-of-fittings, Akaike Information Criterion, visual inspection of the fitted profiles, and CV% of parameter estimates. The ADAPT codes for enacting the mPBPK and PD models are provided in the Supplementary Materials. All figures were created using GraphPad Prism 7.04 (GraphPad Software, La Jolla, CA).

## Results

### Descriptive Summary of the Available Data

The published DEX PK studies found in horses, along with the evaluation of the biomarkers are summarized in Table I. These encompassed four adminstration routes, IV, IM, IA and PO. Part of the data were digitized mean values, while part were the original measurements. The most commonly used dosage of DEX in healthy horses was 0.05 mg/kg across various routes. The horses involved in these studies ranged in age from 2 to 22 years, weighed between 385 and 700 kg, and included females, stallions, and geldings. At least 6 animals were included in each study, supporting the reliability of the mean data. An additional unique study was conducted in horses with LPS-induced joint inflammation to explore the PK/PD of local IA dosing (0.01 ~ 3 mg) under this experimental pathology, with a single animal assigned to each dose level [29]. Across all of these studies, measurements included DEX concentrations in plasma, urine, and synovial fluid, as well as plasma concentrations of CTS and GLU, some of which were sampled over extended periods of up to 4 or 13 days.

**Table I:** Summary of literature data for the pharmacokinetics and pharmacodynamics of dexamethasone (DEX) in horses

Reference	Breed	Age (n, Sex)	Weight (kg)	Dosing Route	Dosage^#^	Clearance (mL/h/kg)	Data Available
Toutain *et al*. 1984 [30]†	Saddlebred	7 ~ 15 yr (1F/5G)	484 ~ 700	IV	(0.05 mg/kg) DEX	768	DEX and CTS in plasma (Start at 7 ~ 9 AM), DEX in urine
				IV	(0.05 mg/kg) DEX_ISO	738	
				IM	(0.05 mg/kg) DEX	NA	
Ekstrand *et al*. 2015, 2019 [36, 37]†	Standardbred	3 ~ 16 yr (6G)	430 ~ 545	IM	0.03 mg/kg DEX_ISO	NA	DEX, CTS, GLU in plasma (Start at 9 AM), DEX in urine, CTS baseline
Ekstrand *et al*. 2019, 2016 [36, 44] †	Standardbred	6 ~ 20 yr (4F/2G)	430 ~ 584	IV	(0.1 µg/kg + 0.0233 µg/kg/h*3 h) DEX_PHO	500	DEX, CTS, GLU in plasma (Start at 9:30 ~ 10:30 AM), CTS baseline
					(1 µg/kg + 0.233 µg/kg/h*3 h)		
					(1 µg/kg + 2.33 µg/kg/h*3 h)		
Authie *et al*. 2010 [6] †	Saddlebred	5 ~ 17 yr (2F/6G)	430 ~ 618	IV	(0.008 mg/kg) DEX	357	DEX in plasma and urine
Soma *et al*. 2005 [7]	Thoroughbred	7.5 ± 1.8 yr (4F/2S)	514.5 ± 51.8	IV	(0.05 mg/kg) DEX	440	DEX, CTS, GLU in plasma (Start at 7 AM)
Soma *et al*. 2013 [39]	Thoroughbred	6 ~ 10 yr (3F/3G)	523.5 ± 44	IV	(0.05 mg/kg) DEX_PHO	450	DEX and CTS in plasma (Start at 8 AM)
				IA	(0.05 mg/kg) DEX_PHO	446	
				IM	(0.05 mg/kg) DEX_PHO	388	DEX in plasma
				oral	(0.05 mg/kg) DEX suspension	446	
Knych *et al*. 2020 [8]	Thoroughbred	4 ~ 7 yr (12)	534 ± 28	IV	40 mg DEX_PHO	473	DEX in plasma and urine
			538 ± 24	oral	(20 mg) DEX tablet		
Haspel *et al*. 2018 [5]	Quarter warmblood	6 ~ 15 yr (1F/5G)	521 ~ 612	IV	5 mg DEX_PHO	463	DEX in plasma
Cunningham *et al*. 1996 [38]	Thoroughbred	2 ~ 5 yr (3F/1S/4G)	396 ~ 476	IV	(10 mg) DEX	479	DEX in plasma
				oral	(10 mg QD*3 days) DEX powder	478	
Grady *et al*. 2010 [2]	Quarter Dutch warmblood	13 ~ 17 yr (4F/2G)	385 ~ 630	oral	(0.05 mg/kg) DEX powder	368	DEX and CTS in plasma (Start at 8 AM), CTS baseline
					(0.05 mg/kg) DEX solution	390	
				IV	(0.05 mg/kg) DEX	434	
Mizen *et al*. 2017 [13]	NA	8 ~ 22 yr (7F/3G)	NA	IM	(0.05 mg/kg QD*7 days) DEX	NA	GLU in plasma
Ekstrand *et al*. 2019 [29]*†	Standardbred	3 ~ 9 yr (3F/3G)	431 ~ 538	IA (with LPS-induced synovitis)	(0.01 mg) DEX_PHO	247	DEX and CTS in plasma (Start at 11:30 AM), CTS baseline
					(0.1 mg) DEX_PHO	266	
					(1 mg) DEX_PHO	446	
					(0.03 mg) DEX_PHO	253	DEX and CTS in plasma (Start at 11 AM), CTS baseline
					(0.3 mg) DEX_PHO	417	
					(3 mg) DEX_PHO	470	

*IV* intravenous, *IM* intramuscular, *IA* intra-articular, *DEX_ISO* DEX isonicotinate, *DEX_PHO* DEX sodium phosphate, *CTS* cortisol, *GLU* glucose

Sex: F = Female, S = stallion, G = gelding. NA, not available

^#^The dosage in parentheses indicates the equivalent DEX dose, while those without parentheses refer to the prodrug

*This study involved horses with lipopolysaccharide (LPS)-induced synovitis, while the others were all conducted in healthy animals

†Data from original studies

### PK of DEX with mPBPK Analysis

**Fig. 2:**
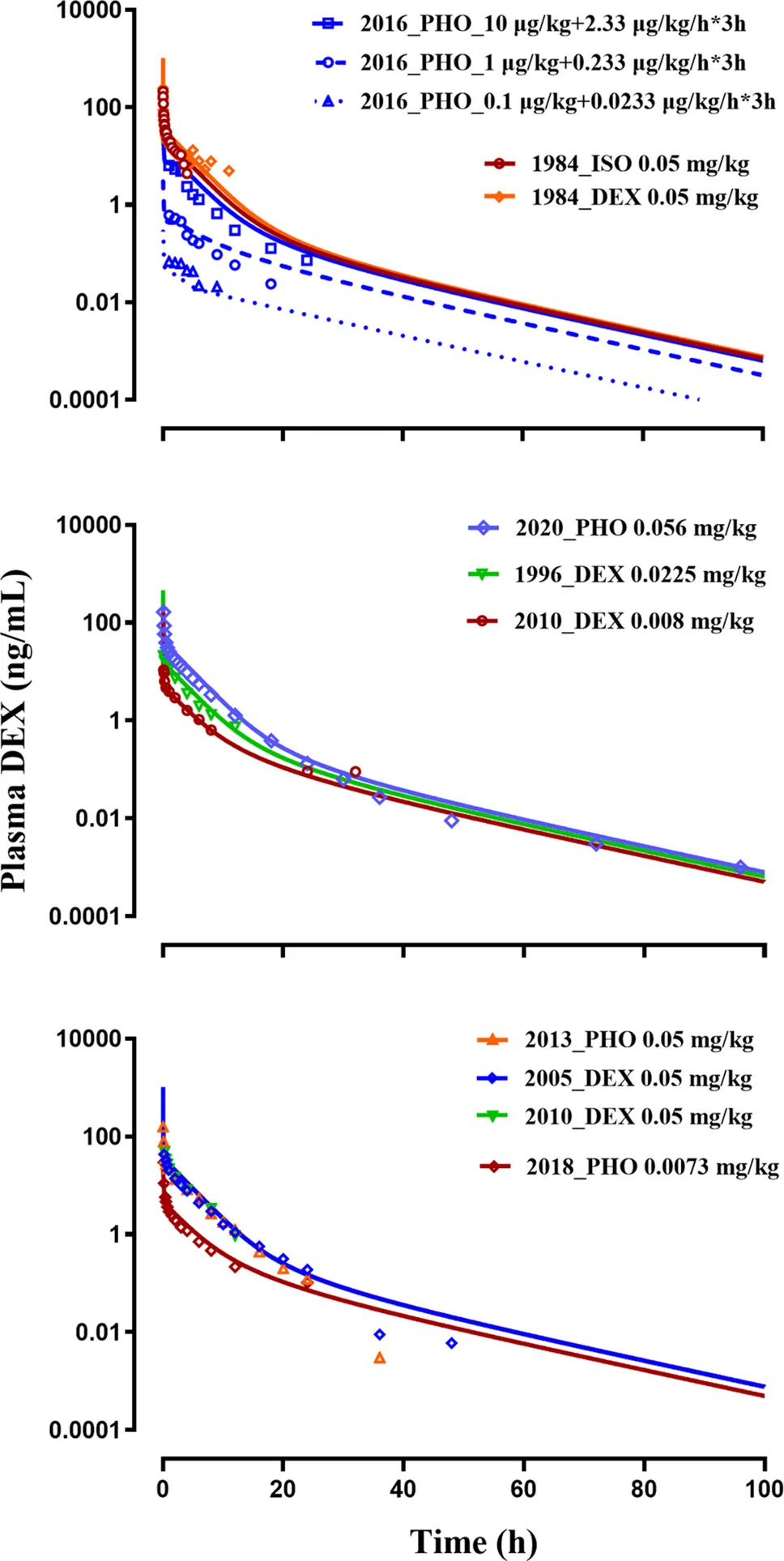
Observed and model-fitted dexamethasone (DEX) plasma concentrations *versus* time following intravenous dosing of DEX alcohol, sodium phosphate (PHO), and isonicotinate (ISO) in healthy horses using the minimal physiologically based pharmacokinetic model. The noted dosages are DEX-equivalent doses of the indicated pro-drugs.

The observed and model-fitted plasma concentration profiles of DEX following IV dosing in healthy horses are shown in Fig. 2. There were 9 PK studies with IV doses of either DEX alcohol, DEX_PHO, or DEX_ISO, covering DEX dosages ranging from 0.1 to 56 µg/kg. The longest sampling period in this group was 96 h. Triexponential kinetics in plasma was observed and well captured by the mPBPK model, which began with a rapid distributive decline, followed by two successive decline phases with decreasing slopes. The mPBPK model parameter estimates obtained from the simultaneous fitting across all dosing routes are listed in Table II. Most CV% are below 35%, indicating the reliability of the estimates. For the commonly used 0.05 mg/kg DEX dose, the half-lifes (*t*_*1/2*_) increased from 2.6 h (5 ~ 15 h phase) to 11 h (2 ~ 4 day phase). The prodrug conversions in blood, for both DEX_PHO and DEX_ISO, were extremely rapid, with estimated *k*_*f*_ of 16 ~ 21 h^−1^ and corresponding *t*_*1/2*_ being less than 3 min. The bioavailability (*F*_*c*_) of IV DEX_PHO fixed to 100% produced good fittings for all six associated profiles, indicating no secondary loss during prodrug conversion. As a result, the plasma concentration–time curve was nearly indistinguishable from that following an equivalent dose of DEX alcohol. DEX_ISO exhibited a 31.5% systemic loss prior to activation, and accordingly slightly lower plasma concentrations were seen compared to DEX alcohol dosing.

**Table II:** Pharmacokinetic parameters of dexamethasone (DEX) using the minimal physiologically-based model

Parameter (units)	Definition		Estimates (CV%)
Prodrug conversion following IV doses			
*k*_*f,VM_i*_ (h^−1^)	Formation rate constant from prodrugs in blood	DEX_PHO	16.67 (34)
		DEX_ISO	20.59 (27)
*F*_*c_i*_ (%)	Prodrug conversion bioavailability	DEX_PHO	100 (fixed)
		DEX_ISO	69.5 (8)
Absorption following IM doses			
*k*_*a,IM_i*_ (h^−1^)	Absorption rate constant from muscle	DEX_PHO	2.95 (56)
		DEX_ISO	0.0105 (5.5)
		DEX	0.131 (19)
Absorption following oral doses in healthy horses			
*k*_*a,PO_i*_ (h^−1^)	Oral absorption rate constant	Powder/tablets	0.421 (11)
		Suspension/solution	0.529 (11)
*F*_i_* (%)	Total oral bioavailability	Powder/tablets	37.2
		Suspension/solution	48.7
*F*_*GI_i*_ (%)	Gastrointestinal tract bioavailability	Powder/tablets	52.1 (8)
		Suspension/solution	68.2 (9)
Prodrug conversion and absorption following IA doses			
*k*_*f,IA_PHO*_ (h^−1^)	Formation rate constant from DEX_PHO in joints		30 (fixed)
*k*_*a,IA*_ (h^−1^)	Absorption rate constant from healthy joints		2.61 (24)
	Absorption rate constant from inflamed joints		5.13 (7)
*F*_*IA*_ (%)	Bioavailability following IA injection into healthy joints		83.0 (11)
	Bioavailability following IA injection into inflamed joints		100 (fixed)
Shared drug disposition across all administration routes			
*B*_*max*_ (ng/mL)	DEX binding capacity in liver		140.1 (12)
*K*_*d*_ (ng/mL)	Equilibrium dissociation constant of DEX		0.269 (16)
*K*_*p,r*_	DEX partition coefficient		1.09 (5)
*CL*_*h,int*_ (mL/h/kg)	Intrinsic hepatic clearance		473.6 (5)
*CL*_*r*_ (mL/h/kg)	Renal clearance		5.69 (10)

*Calculated as the ratio of the areas under the predicted plasma concentration–time curves following oral and IV administration at the same dosage (*AUC*_*PO*_/*AUC*_*IV*_)

**Fig. 3:**
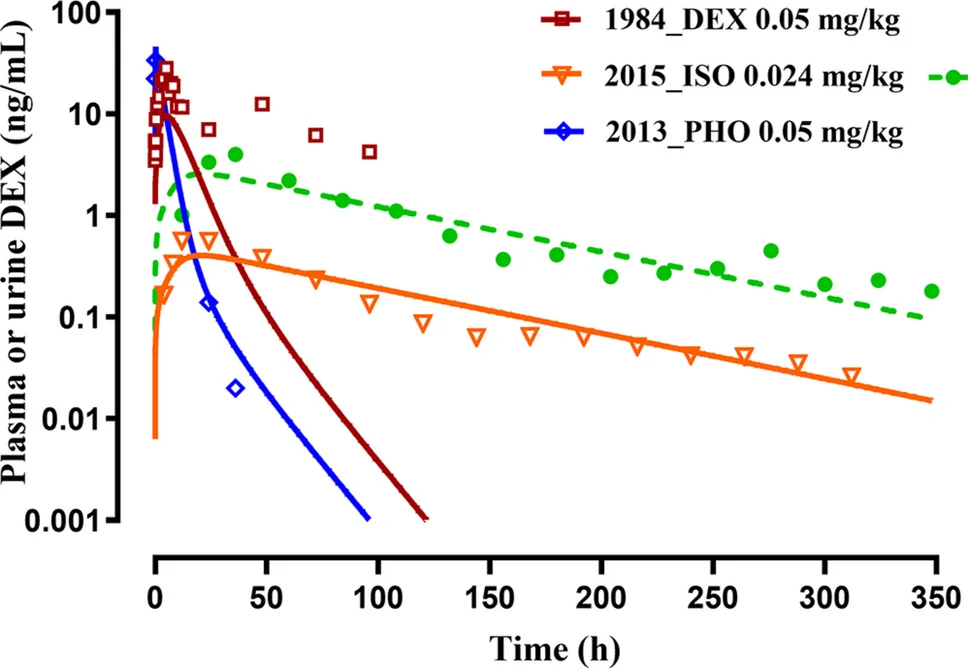
Observed and model-fitted DEX concentrations in plasma and urine following intramuscular administration of DEX alcohol, sodium phosphate (PHO), and isonicotinate (ISO) in healthy horses. Urine concentrations are shown as green symbols and dashed line

The observed and model-fitted plasma and urine concentration profiles following IM doses are shown in Fig. 3. It included three studies, covering three DEX formulations and two dosages, 0.024 and 0.05 mg/kg. One of the studies collected both plasma and urine for up to 14 days. The plasma data following DEX alcohol dosing were poorly captured by the model, whereas the remaining profiles were well described. The IM absorption rate constants (Table II) varied markedly among the three forms: DEX_PHO exhibited the fastest absorption (*k*_*a*_ = 2.95 h^−1^, *t*_*1/2*_ = 14 min), followed by the free alcohol form (*k*_*a*_ = 0.131 h^−1^, *t*_*1/2*_ = 5.3 h), and DEX_ISO the slowest (*k*_*a*_ = 0.0105 h^−1^, *t*_*1/2*_ = 67 h). These differences are clearly reflected by their corresponding peak times (*t*_*max*_), 0.23, 4.7, and 21 h in Fig. 3. Upon IM absorption, both DEX alcohol and DEX_PHO exhibited similar profiles to those following IV doses, with presumed rapid hydrolysis of the pro-drug ester and negligible absorption loss. In contrast, the plasma profile following IM DEX_ISO dosing exhibited biexponential kinetics and a prolonged terminal phase. The terminal *t*_*1/2*_ was 67 h, identical to the absorption *t*_*1/2*_ and indicative of typical flip-flop kinetics. The corresponding urinary profiles paralleled the plasma profiles, suggesting passive and linear renal clearance. The plasma concentration of DEX following IM dosing of DEX_ISO (0.024 mg/kg DEX) remained twofold above a 5 pg/mL threshold at the end of the 15-day study. In an early study, a 0.05 mg/kg IM dose of DEX_ISO was given, but DEX plasma concentration were undetectable with the methods available at the time [30].

**Fig. 4:**
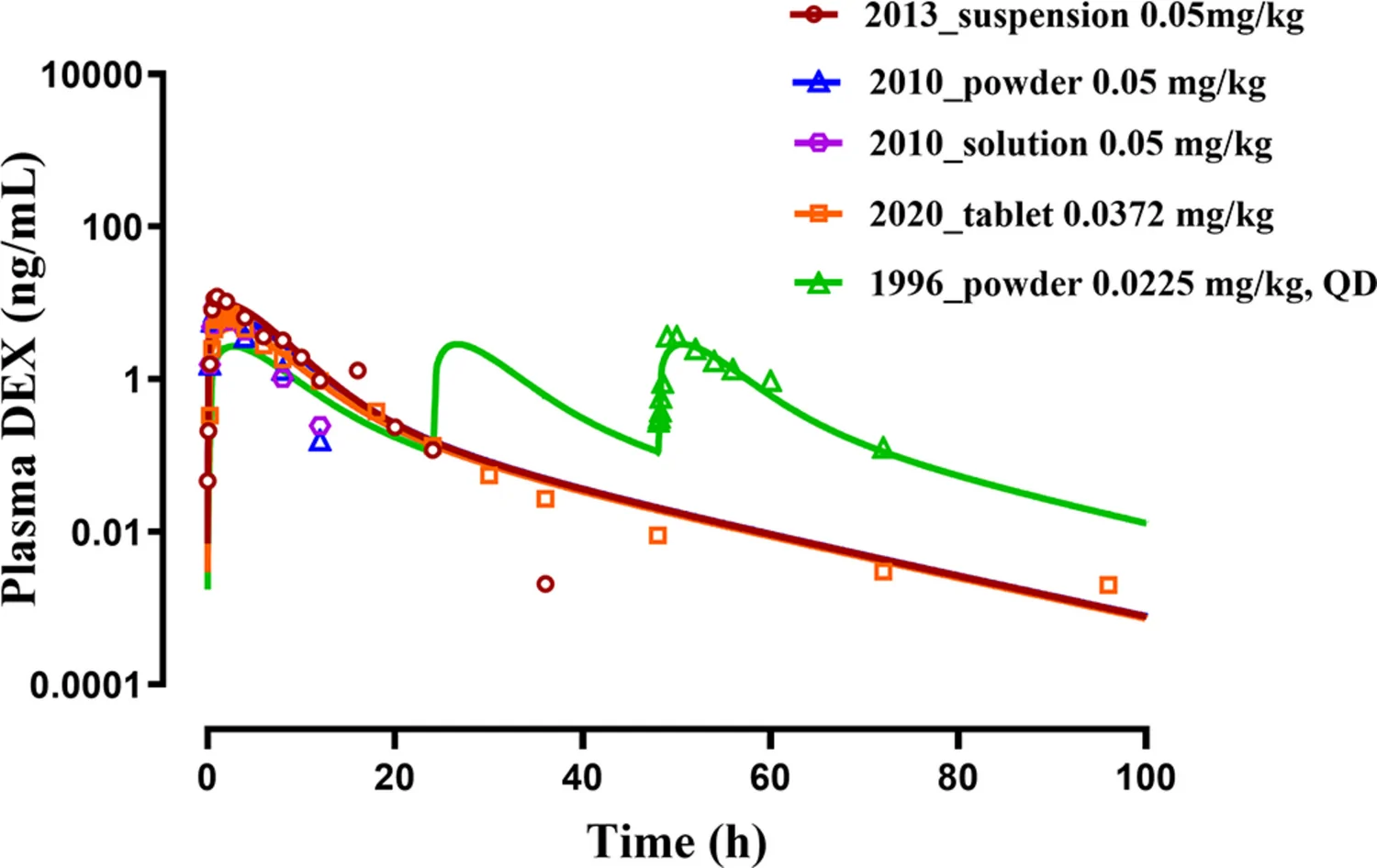
Observed and model-fitted DEX concentrations in plasma following oral administration of DEX in healthy horses

The observed and model-fitted plasma concentration profiles following oral doses are shown in Fig. 4 based on four single-dose studies and one multiple-dose study. Four different physical forms of DEX were investigated: injectable DEX suspension and solution, intact tablet, and tablet powder, with doses ranging from 0.0225 to 0.05 mg/kg. All observed and model-predicted PO profiles showed good agreement. Triphasic kinetics was observed with a moderate input rate and biexponential decline. The *t*_*1/2*_ of the two declining phases were identical to those after IV administration. The oral absorption *F*_*GI*_ was estimated at 52.1% for the solid powder or tablet and 68.2% for the liquid solution or suspension. Notably, these values reflect partial bioavailability, as they do not account for hepatic first-pass metabolism that was built into the model. The complete *F*, calculated as the ratio of the areas under the predicted plasma concentration–time curves following oral and IV administration *(AUC*_*PO*_/*AUC*_*IV*_) over a 20-day period, were 37.2% for the solid drugs and 48.7% for the liquids. The *k*_*a*_ was similar for solid (0.42 h^−1^) and liquid forms (0.53 h^−1^). Their *t*_*max*_ was predicted at 2.2 and 2.7 h, which were earlier than the IM DEX alcohol (4.7 h). Similarly, Krzyzanski *et al*. [31] also found that oral DEX is absorbed faster than IM DEX in humans.

**Fig. 5:**
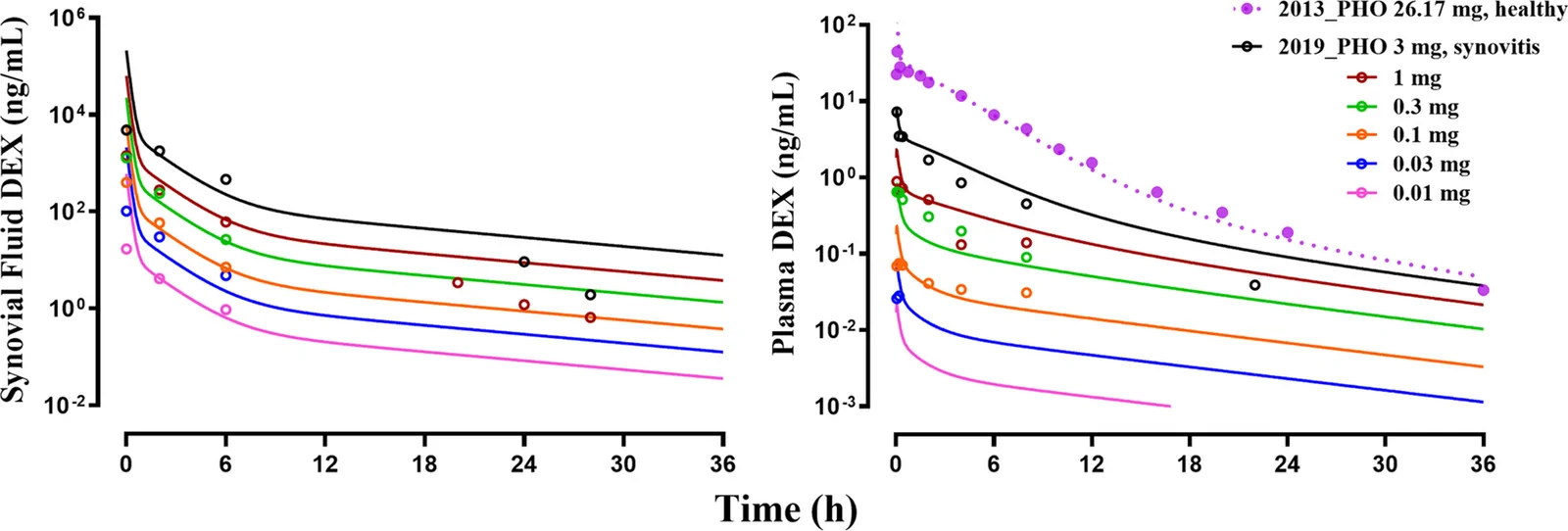
Observed and model-fitted DEX concentrations in plasma and synovial fluid following intra-articular injection of DEX sodium phosphate (PHO). Purple symbols and dashed line depict PK profiles following IA injection in healthy joints, while the remaining profiles correspond to joints with LPS-induced synovitis

The observed and model-fitted DEX concentration profiles in SF and plasma following IA dosing are shown in Fig. 5. It included 1 single-dose of 26.17 mg (0.05 mg/kg) DEX_PHO in healthy joints and 6 single-doses, 0.01 ~ 3 mg DEX_PHO, injected in inflamed joints. These digitized mean data were successfully captured by the model. In plasma, DEX concentrations upon IA injection in both healthy and inflamed joints followed a triexponential decline, without a visible absorption delay. Except during the first 10 min, the model-predicted curves for IA and IV dosing at 0.05 mg/kg were nearly superimposable, as IA absorption was immediate, rapid, and extensive with an estimated *k*_*a*_ of 2.64 h^−1^ (*t*_*1/2*_ = 0.26 h) and *F* of 82.5% in healthy joints. By comparison, DEX absorption from inflamed joints was more rapid and complete, with *k*_*a*_ of 5.16 h^−1^ (*t*_*1/2*_ = 0.13 h) and (assumed) *F* of 100%. Additionally, DEX was rapidly formed from DEX_PHO in the inflamed joint, the *k*_*f*_ was fixed at 30 h^−1^ due to consistent convergence to this value during preliminary model development and its practical unidentifiability. DEX exhibited a triexponential decay expressed using Eq. 7 with *f*_*i*_ (i = 1, 2, 3) = 0.9783, 0.02098, and 0.0007512, *α* = 6.354 h^−1^, *β* = 0.5977 h^−1^, and *γ* = 0.07056 h^−1^. This indicates that most of the dose in the joint (97.8%) disappeared rapidly with a *t*_*1/2*_ of 0.11 h, quickly appearing in plasma (*k*_*a*_ = 5.16 h^−1^).

DEX was modeled as primarily eliminated via hepatic metabolism, with the estimated systemic hepatic clearances (*CL*_*h*_) and extraction ratio (ER = *CLh/Ql*) being 338 mL/h/kg and 28%, which are consistent with literature reports of 328 mL/h/kg and 27% [6]. The renal clearance (*CL*_*r*_) was 5.69 mL/h/kg, which is lower than the predicted glomerular filtration clearance (*CL*_*GF*_) of 18.4 mL/h/kg, as the product of unbound fraction (0.175) [21] and equine glomerular filtration rate (105 mL/h/kg) [32]. This suggests that DEX is both filtered by the glomeruli and subject to passive tubular reabsorption. The total *CL* of DEX in horses was 344 mL/h/kg, falling within the reported *CL* range of 247 ~ 768 mL/h/kg shown in Table I. The estimated hepatic binding capacity (*B*_*max_l*_) of DEX was 140.1 ng/mL, which is lower than that reported for methylprednisolone (MPL) (322.9 ng/mL) [22] and prednisolone (188.5 ng/mL) [33] in rats, suggesting a potentially lower GR density in equine liver. The equilibrium dissociation constant (*K*_*d*_) of DEX in liver was 0.27 ng/mL, which is lower than that of MPL at 0.74 ng/mL in horses, indicating a higher affinity of DEX. This finding is consistent with the stronger receptor binding of DEX compared to MPL.

**Fig. 6:**
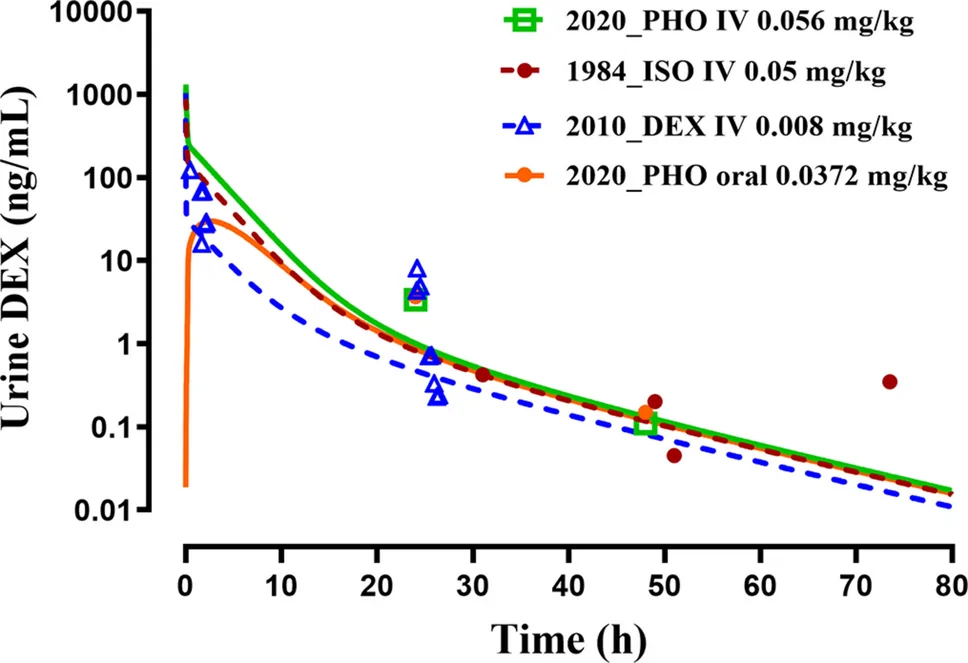
Observed and model-simulated dexamethasone (DEX) concentrations in horse urine following intravenous (IV) and oral doses. Symbols are available observations and lines are model simulated profiles. The noted dosages are DEX-equivalent doses of the indicated pro-drugs

The sparse observations and model-simulated DEX concentration profiles in urine are shown in Fig. 6. The strong agreement suggested that the PBPK model performed very well in predicting urinary DEX concentrations across the four studies.

### Adrenal Suppression Dynamics

**Fig. 7:**
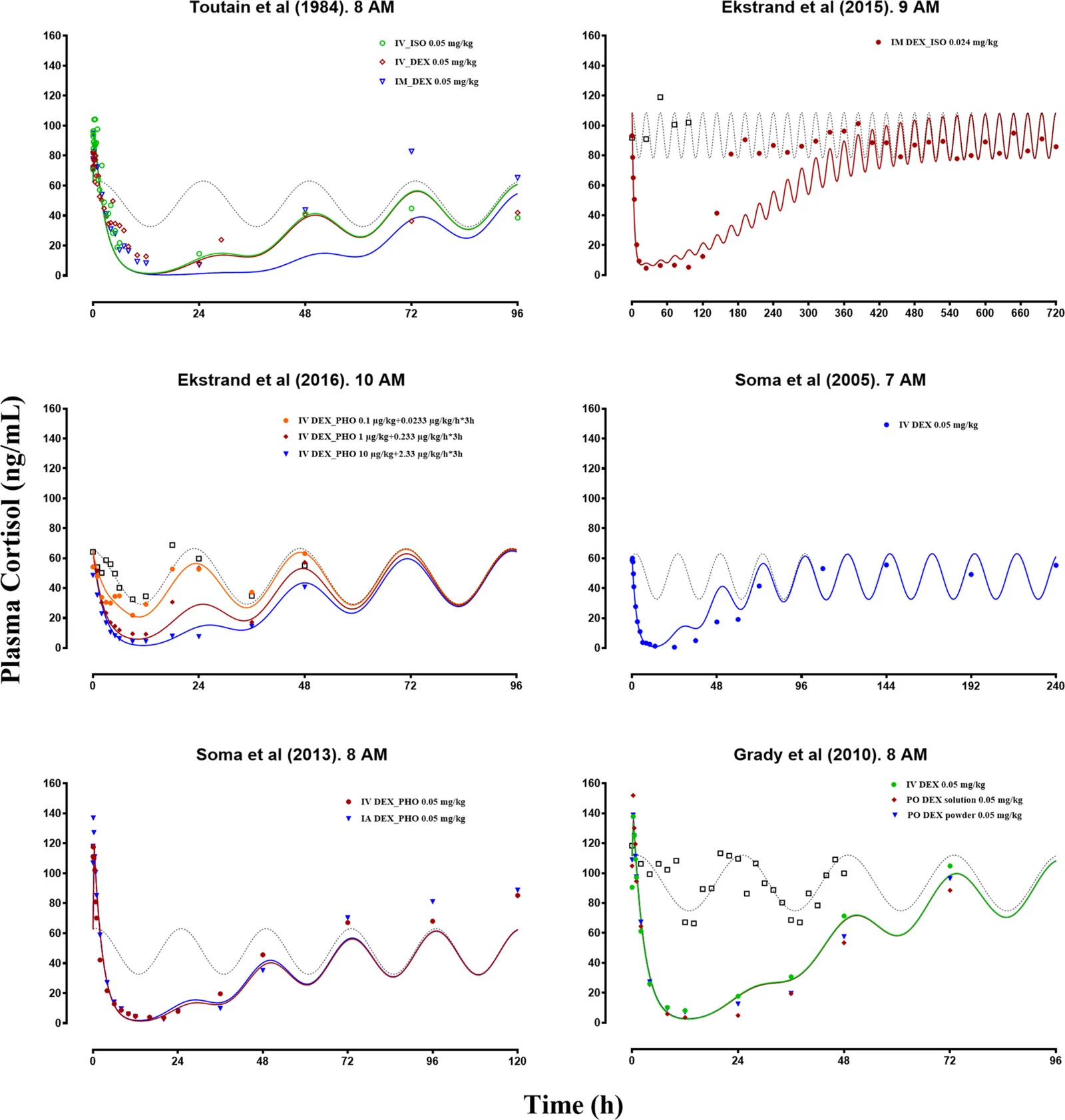
Observed and model-fitted plasma cortisol concentrations *versus* time in horses. Symbols depict observations (mean values), solid lines are model-fitting results after dexamethasone (DEX) doses. The black squares and dashed lines depict the cortisol baseline rhythm. The dosages are DEX-equivalent doses of the indicated pro-drugs. The clock time corresponding to time zero of the PK studies is indicated in the panel titles.

The observed and model-fitted CTS concentration–time profiles after DEX dosing are shown in Fig. 7. Overall, the proposed model captured the CTS baseline and drug effects reasonably well, with baseline- and drug-specific parameters listed in Table III. Without drug, the basal plasma CTS concentrations were in a typical circadian rhythm. They exhibited a peak time occurring at 8:56 AM, a study-dependent mesor (mean), 47.89 ~ 93.42 ng/mL, and a study-dependent amplitude, 15.23 or 22.01 ng/mL. The estimated input acrophase (8:56 AM) aligns with the observed cortisol peak at 9:11 AM, as reported in an intensive temporal sampling study involving 10-min intervals [24]. The CTS loss rate constant (*k*_*out_CTS*_) was 0.41 h^−1^, which closely agrees with the reported range, 0.27 ~ 0.46 h^−1^, based on a similar mechanism-based model [34]. This indicates that the *t*_*1/2*_ of CTS in horses is 1.7 h, which is consistent with the reported terminal *t*_*1/2*_ of 2.19 h from a PK study involving exogenous hydrocortisone administration [25].

**Table III:** Pharmacodynamic parameters for dexamethasone altering plasma cortisol (CTS) and glucose (GLU) responses

Parameter	Definition	Estimates (CV%)
Cortisol		
*T* (h)	Circadian period	24
*R*_*a*_ (ng/mL)	Amplitude	15.23 (16)^a,b^/22.01 (14)^c^
*R*_*m*_ (ng/mL)	Mean baseline or mesor	93.42 (2.2)^a^/62.25 (4.8)^c^/47.89 (6.5)^b^
*t*_*p*_ (h)	Acrophase expressed as clock time	8:56 AM (61)
*k*_*out_CTS*_ (h^−1^)	Loss rate constant for CTS	0.405 (4.9)
*R*_*max,stress*_ (ng/mL)	Stress-induced maximal CTS surge	44.76 (9)^d^/68.09 (13)^e^/38.73 (9)^a^
*τ* (h)	Duration of CTS surge	0.34 (fixed)
*I*_*max*_	Maximal inhibitory index	1.0 (fixed)
*IC*_*50*_ (ng/mL)	DEX concentration at 50% maximal inhibition	0.0383 (8.9)
Glucose		
*E*_*0*_ (mM)	Baseline GLU in plasma	4.65 ~ 5.47 (1.9 ~ 3.1)
*k*_*in_GLU*_ (mM/h)	Zero-order production rate constant of GLU	0.225 ~ 0.265 (2.0 ~ 4.7)
*k*_*out_GLU*_ (h^−1^)	Loss rate constant for GLU	0.0485 (17.8)
*E*_*max*_	Maximal stimulatory index	1.187 (51)
*EC*_*50*_ (ng/mL)	DEX concentration at 50% maximal stimulation	0.799 (21)

^a^For Grady *et al*. 2010 [2]. ^b^ Ekstrand *et al*. 2015 [37]. ^c^ Ekstrand *et al.* 2019 [29]. ^d^For Toutain *et al*. 1984 [30]. ^e^For Soma *et al*. 2013 [39]

DEX caused rapid and profound adrenal suppression, reducing CTS concentrations to almost zero (~ 1 ng/mL), thereby justifying the fixed *I*_*max*_ value of 1 in the model. Following single doses, the maximal suppression occurred at a time coinciding with the earliest and lowest point in the circadian cycle, and CTS concentration progressively approached the baseline thereafter. Regardless of DEX dosage, it took 3 ~ 4 rhythmic cycles for CTS to recover to the baseline. There were minimal differences in CTS suppression observed among IV, IA, and oral doses, whereas IM dosing of DEX_ISO with its prolonged PK suppressed CTS for a significantly longer duration. The corresponding *IC*_*50*_ was estimated at 0.038 ng/mL, identical to a reported value of 0.037 ng/mL [34]. This is approximately 20-fold lower than that of MPL for adrenal suppression in horses, 0.83 ng/mL [18], and fourfold lower than that of DEX in humans, 0.14 ng/mL [35]. Additionally, dose-dependent adrenal suppression by DEX is seen in Fig. 7. In some studies with intensive post-dose sampling, stress-induced CTS surges were seen during the initial phase. The magnitude of this response varied across studies, with CTS increased by 39 ~ 68 ng/mL within 20 min after the start of the study.

**Fig. 8:**
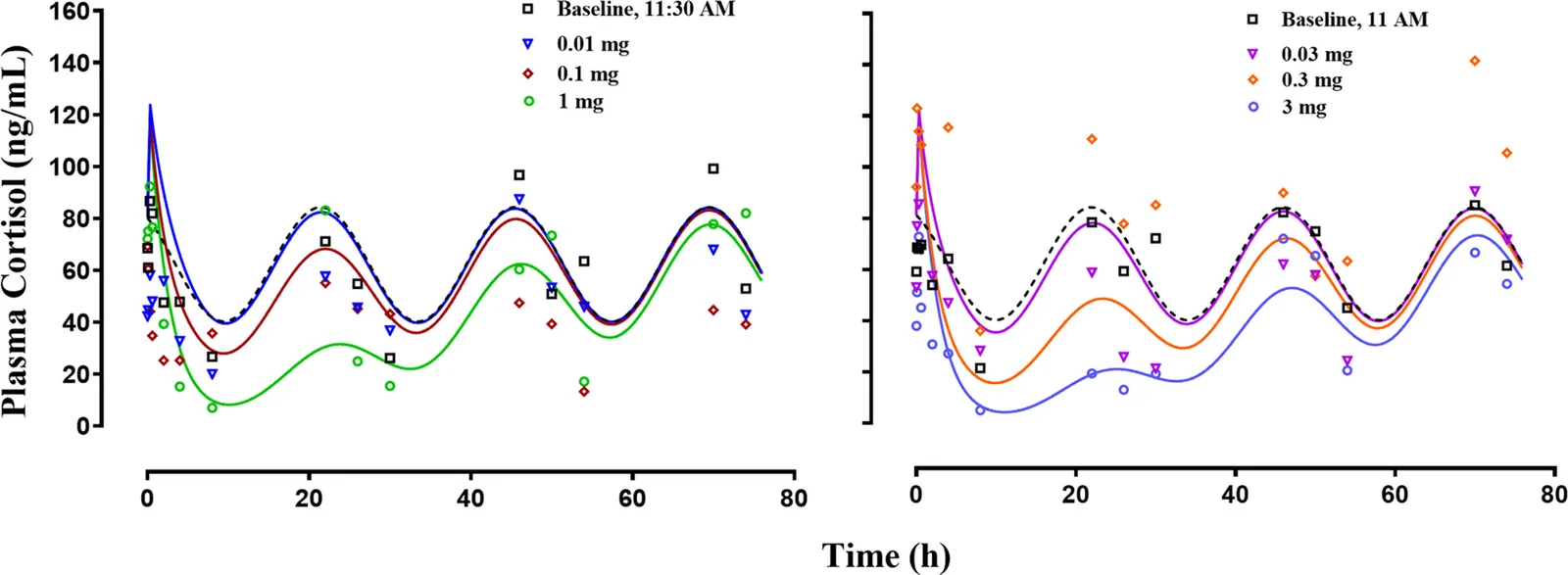
Observed and model-simulated cortisol concentrations in horses following intra-articular injections of dexamethasone sodium phosphate. Symbols depict measurements, solid lines are model-predictions, and dashed lines depict the fitted baseline. The noted dosages are DEX-equivalent doses administered

The observed and model-simulated CTS responses following a series of IA doses are shown in Fig. 8. In this simulation, the inflamed joint-specific absorption parameters (*k*_*a*_ = 5.16 h^−1^, *F* = 100%), study-specific CTS baseline parameters (*R*_*a*_ = 22.01 ng/mL, *R*_*m*_ = 62.25 ng/mL), a mean *R*_*max,stress*_ of 50.53 ng/mL, and other PK and PD parameter values listed in Tables II and III were applied. Except for the 0.3 mg dose, which had unexplained high variability, all other fitted CTS concentration–time curves achieved good concordance with the experimental data.

### Plasma Glucose Dynamics

**Fig. 9:**
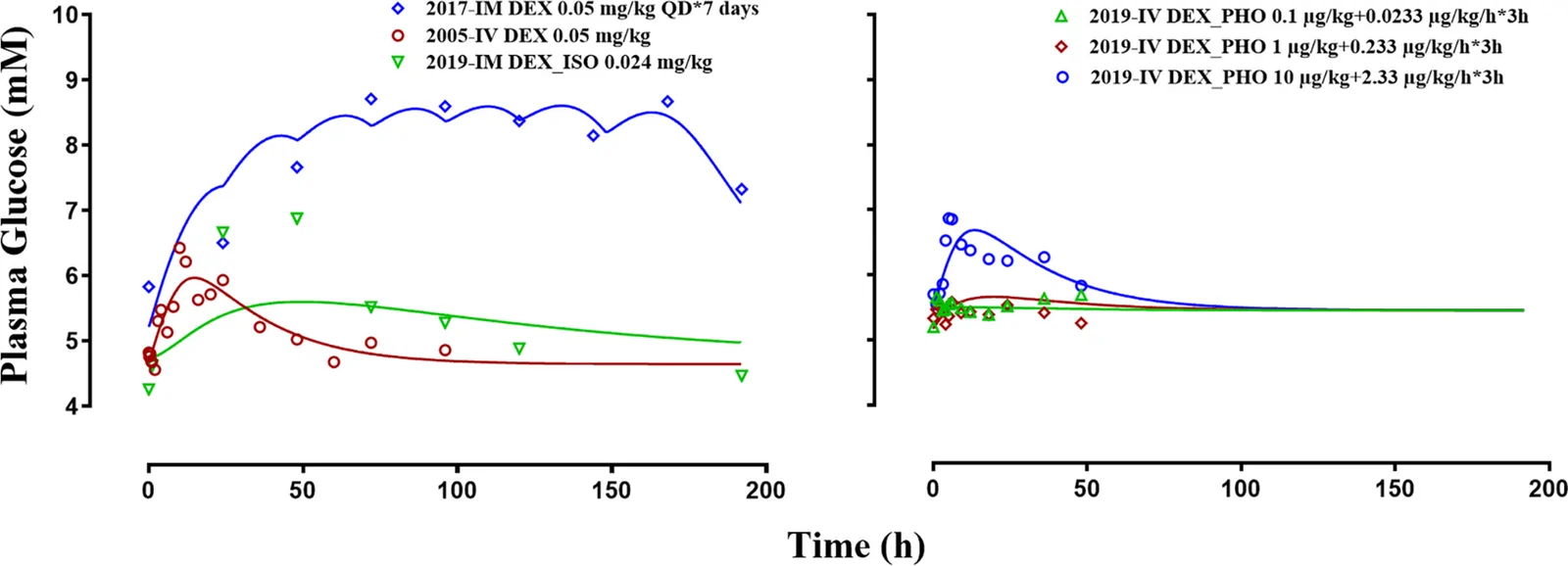
Observed and model-fitted plasma glucose concentrations *versus* time. Symbols depict digitized observations, solid lines are model-fitting results after dexamethasone (DEX) doses, and broken lines depict the baselines. The noted dosages are DEX-equivalent doses.

The observed and model-fitted plasme GLU concentration–time profiles following DEX dosing are shown in Fig. 9. Following a single IV dose of DEX, peak GLU occurred around 14 h post-dose and returned to baseline in 2 days. The increases were very small with the single doses of DEX. With daily IM doses of 0.05 mg/kg DEX, GLU remained elevated at approximately twice the baseline, reaching ~ 8.5 mM. The PK/PD model adequately captured the DEX-induced plasma GLU changes, with relevant PD parameters listed in Table III. Individual plasma GLU baselines ranged from 4.65 to 5.47 mM. According to this model, GLU was produced in horses at a constant rate of ~ 0.25 mM/h and dissipated with a *t*_*1/2*_ of 14.3 h. This most likely reflects enzymatic turnover in the liver rather than insulin-induced tissue uptake.

The maximal stimulatory index of DEX (*E*_*max*_) on GLU production was 1.187 (CV% = 51%). Accordingly, the expected maximal GLU concentration could theoretically reach 11.24 mM with a sufficiently high DEX dose. The *EC*_*50*_ was 0.80 ng/mL, substantially higher than the *IC*_*50*_ (0.038 ng/mL), indicating lower gluconeogenic sensitivity to DEX. Notably, this *EC*_*50*_ confirms a literature value of 0.62 ng/mL based on a similar PD model [36]. In humans, the *EC*_*50*_ for the same response was 17.9 ng/mL [31], suggesting greater gluconeogenic sensitivity to DEX in horses.

## Discussion

This report provides a comprehensive system pharmacology model of DEX in horses that concurrently integrates drug input, tissue distribution, adrenal suppression, and glycemic effects within a physiologically informed framework. The PK of three DEX formulations given via four different routes were compared to explore the convergence in systemic drug disposition and divergence in pre-systemic prodrug conversion and absorption. The effects of DEX on adrenal suppression and gluconeogenesis were evaluated, focusing on the onset, extent and duration of both responses, as well as the drug-free baselines. Model development first proceeded with the majority of datasets and then the established models were used to predict expectations in the remaining datasets conducted for special purposes or being of lower quality, as an internal validation. The derived PK/PD parameters of DEX were compared with those reported in literature for DEX and other GCs to demonstrate model consistency. The model-predicted DEX exposures and durations can be compared with the regulatory thresholds or limits adopted by various jurisdictions to demonstrate the usefulness gained by this comprehensive assessment. There was great consistency in the PK/PD data across the many studies (Table I) that facilitated this meta-analysis.

Our PBPK model to analyze PK data for DEX in horses differs from earlier studies. Previously, various mammillary models were employed, ranging from one- [37, 38], two-[34, 38], to three-compartment models [8, 39], with model selection largely driven by the duration of blood sampling. Our PBPK model is mechanistic and allows a comprehensive and unified assessment, as evidenced by the strong agreement between observed and model-predicted profiles in Figs. 2, 3, 4, 5 and 6. The PK profiles of DEX in plasma and urine exhibit three phases, featuring a previously unappreciated prolonged terminal elimination phase. Along with extending previous DEX PK models for rats [40] and across species [41], this model distinguishes and quantifies hepatic and renal elimination pathways owing to the availability of urine data, inclusion of the liver, and availability of oral dose data.

In horses, DEX primarily undergoes elimination in the liver and kidneys, with hepatic metabolism accounting for approximately 98% of the total clearance and renal excretion contributing the remaining 2%. These proportions closely match previously reported values of 95% *versus* 5% [6] and are also similar to results for MPL, 93% *versus* 7% [18]. The renal excretion of DEX involves both glomerular filtration and tubular reabsorption, consistent with its small molecular size (MW = 392) and moderate lipophilicity (log *P* = 1.83). In dogs [42] and rabbits [43], dose-dependent plasma PK of DEX was observed. In contrast, linear PK has been consistently reported across multiple studies in rats, horses and humans [44–47]. However, our model incorporated saturable tissue binding in liver, which theoretically results in a decrease in the apparent *CL* as the dose increases and producing a time-varing terminal *t*_*1/2*_. Across the six IA doses evaluated (Fig. 5), typical target-mediated drug disposition-like features were observed in plasma, such as a steeper distributive phase at lower doses and parallel terminal phases [48]. These phenomena were also evident in the IV dose profiles in Fig. 2. Further support is found in an *in vitro* liver homogenate binding assay [21], where DEX showed a higher bound fraction at 1 than at 10 μg/mL, consistent with saturable binding. With the constant renal clearance, urine and plasma concentration profiles of DEX declined in parallel. The urine-to-plasma concentration ratio (*R*_*u/p*_) of DEX remained constant over time at 6.3, which is the theoretical value of *CL*_*r*_/*V*_*u*_ and comparable to the reported ratio of approxinately 10 [37].

The three formulations involved in this study, DEX_ISO, DEX_PHO, and alcohol forms, differ in physicochemical properties, which in turn influence their absorption. DEX_ISO, due to its very poor water solubility [37], is formulated as an injectable suspension and commonly used for depot administration. This pro-drug was dissolved in propylene glycol for the IV dosing study [30]. The slow dissolution of this “nearly insoluble” prodrug resulted in a sustained-release input and flip-flop kinetics after IM dosing, as shown in Fig. 3. This is futher supported by the extremly low *k*_*a*_, 0.01 h^−1^, where the absorption rate became the rate-limiting step. In contrast, DEX_PHO is highly water-soluble [30, 49] and was rapidly absorbed. Our modeling of the pre-systemic processes, namely prodrug conversion and absorption, aligns closely with known physiological and anatomical principles. Carboxylesterase, the key enzyme responsible for prodrug hydrolysis, is abundantly expressed in the liver [50], is present at high content in plasma in horses but not in humans [51], and is negligible in muscle [52]. This justified the assignment of DEX activation in blood following IV and IM doses and the immediate availability of IV DEX from either ester formulation. Regarding IA doses, there was rapid local DEX appearance from DEX_PHO in SF (Fig. 5). Carboxylesterase activity in SF was demonstrated previously by the rapid hydrolysis of MPL acetate to MPL in an *in vitro* study using fresh bovine SF [53]. Different modeling approaches were adopted for healthy and inflamed joints primarily due to varied types of data availability. Simplified differential equations were applied to healthy joints to capture the essential prodrug conversion and DEX absorption processes as evident in plasma, whereas empirical triexponential functions were employed for inflamed joints to reflect the available synovial fluid concentration–time profiles as well as drug exposure in plasma.

Oral does of DEX undergo sequential losses due to incomplete absorption (slow dissolution, or local degradation) and then the intestinal or hepatic first-pass effect. To mimic this pathway, oral absorption was modeled by linking the GI absorption site to the liver. This approach yielded *F*_*GI*_ values of 68.2% for the liquid form and 52.1% for the solid form, reflecting incomplete GI absorption and/or potential intestinal metabolism. The AUC-based *F* values of 48.7% and 37.2% reflect the overall systemic availability and fall midrange to reported oral dose *F* values: 31% [2], 31 ~ 88% [38], 52.6% [39], and 61% [8]. The hepatic bioavailability (*F*_*H*_ = *F*/*F*_*GI*_), or proportion escaping first-pass, was accordingly 71.4%, which is consistent with the hepatic ER of 28%. The CYP3A enzymes primarily responsible for DEX metabolism [54] are found in both the intestine and liver [55]. The partial bioavailability of oral DEX in horses contrasts with the apparent near full bioavailability in humans [56].

The PD model describing cortisol dynamics extends an existing model [44], where the oscillatory cortisol baseline and IDR model had been applied. Population-based analyses evaluating the cortisol response following IV and PO DEX in horses have also been reported [8, 34]. Our model added the early stress-induced irregular fluctuations. Cortisol in horses can be expected to be disrupted by acute stressors as part of the "fight or flight" response triggered by injection and sampling pain, handling, or physical restraint. Some studies missed this transient response by not sampling during the first 30 min [37, 44] or by minimization of external stimuli through the use of skilled technicians and acclimatization training [7]. Based on the 15 ~ 30 min CTS surge observed by Toutain *et al*. [24], the duration of this stress-induced response was fixed at 0.34 h (20 min) in our model. Its extent varied across studies and the average *R*_*max,stress*_ of 50.53 ng/mL was applied for IA doses. Besides the intial CTS surge, the *R*_*m*_ and *R*_*a*_ relevant to CTS baseline also exhibted high varibilities across studies and even among individuals. Such concentrations are influenced by physiological and environmental factors, including breed, age, sex, daily routine, and season [57–59]. Therefore, study-specific *R*_*m*_ and *R*_*a*_ parameters were applied in modeling and simulations when drug-free control data were available. Otherwise, visually matched baseline parameters from comparable studies were assigned. The presumed stress-induced early surge in cortisol is obviously a complication if cortisol is used as a biomarker to assess DEX effects.

Our PD model used the mPBPK-derived plasma concentrations of DEX to drive drug effecs. As expected, the estimated *IC*_*50*_ of DEX (0.038 ng/mL) agreed with the reported range of 0.007 ~ 0.06 ng/mL from other PD models [8, 34, 37, 44], wether considering the oscillating CTS baseline or not. Concerns existed regarding the presence of a distinct circadian rhythm of CTS in horses, with some studies reporting clear rhythmicity, while others failed to detect such patterns. This controversy was subsequently addressed by a study demonstrating that suboptimal experimental conditions, such as insufficient habituation, likely masked the underlying circadian rhythm [60]. Our study jointly assessed the adrenal suppression by DEX regardless of dosing routes (except for IM DEX_ISO), marked by a rapid CTS decline within 12 h following all morning doses and a gradual recovery returning to basline over 3 ~ 4 days. When interpreting the delayed return of CTS to baseline, Soma *et al*. [7] proposed the presence of a sustained tissue reservoir that allowed DEX to persist in peripheral tissues for up to 3 ~ 4 days following a single bolus dose. This is essentially our high-affinity tissue binding component (*Bmax*) in liver. Our linked mPBPK/PD model demonstrated reasonable predictions of DEX concentrations in urine (Fig. 6), the most-common biofluid for anti-doping control, and cortisol concentrations in plasma (Fig. 8), a recognized biomarker of DEX effects.

The glucose response model was semi-mechanistic in nature, focusing solely on the predominant effect of DEX-induced hepatic gluconeogenesis. It lacked a placebo study and inclusion of possible food effects. The turnover components of the current glucose model likely reflect induction of phosphoenolpyruvate carboxykinase (PEPCK) mRNA and protein in the liver as directly observed for MPL in rat liver [61]. Ideally, DEX concentrations binding to GR in liver, rather than plasma concentrations, should serve as the authentic driving force for gluconeogenesis. However, the model fitting was satisfactory as enacted here and done previously in humans [31].

Given the strict regulation of equine DEX use and advancements in analytical techniques, many regulatory authorities have established thresholds to distinguish legitimate therapeutic administration from potential misuse. In addition to the commonly adopted plasma threshold of 5 pg/mL [9, 10], the harmonized screening limits (HSL) for DEX in urine is set at 0.2 ng/mL by the International Federation of Horseracing Authorities (IFHA) [62]. To guide trainers and veterinarians in adhering to these regulations, European Horserace Scientific Liaison Committee (EHSLC) [63] and IFHA [64] have published detection times (DTs): 5 days for 0.06 mg/kg IV DEX_PHO and 14 days for 0.03 mg/kg IM DEX_ISO. Additionally, the Kentucky Horse Racing Commission (KHRC) mandates a withdrawal time (WT) of 72 h and a dosage limit of 0.05 mg/kg for oral, IV or IM DEX administration [65].

This study offered quantititave information and supporting evidence in relation to regulatory rules. Taking 0.05 mg/kg as the reference DEX dose, the plasma concentrations of DEX declined to the ARCI threshold (5 pg/mL) at 67 h regardless of route (except for IM DEX_ISO), which is comparable to the WT time enforced by the KHRC, 72 h. Toutain and Lassourd [66] proposed calculations for “irrelevant” plasma (IPC) and urine concentrations (IUC) for drug control in horses as: IPC = [standard dose per dosing interval/(*CL*· Safety Factor)] and IUC = IPC ·* R*_*u/p*_, below which a drug can be viewed as absent of any relevant drug effect or need for regulatory action. For DEX in horses, the IPC and IUC were estimated to be 4 and 25 pg/mL, using a dosage of 0.05 mg/kg, dosing interval of 72 h, *CL* of 344 mL/h/kg, default Safety Factor of 500, and *R*_*u/p*_ of 6.3. The resulting IPC (4 pg/mL) was close to the ARCI plasma threshold (5 pg/mL) and tenfold lower than the *IC*_*50*_ for adrenal suppression (38 pg/mL), which is considered reasonable.

On the other hand, the ARCI plasma threshold of 5 pg/mL would correspond to an concurrent urine threshold of 31 pg/mL as *R*_*u/p*_ was 6.3. According to our model (Fig. 3), urinary DEX concentrations declined to the IFHA issued HSL (0.2 ng/mL) at approximately 11.5 days following a 0.03 mg/kg IM dose of DEX_ISO, which is slightly shorter than the IFHA/EHSLC-proposed 14-day detection time. Similarly, after a 0.06 mg/kg IV dose of DEX_PHO (Fig. 6), urinary concentrations fell below the HSL within 40 h—considerably shorter than the 5-day DT. As a result, the withdrawal times would be 20 days and 72 h, representing an approximate 1.8-fold extension (uncertainty factor range: 1.4 ~ 2.2) relative to the DTs, as suggested by Toutain [67]. The latter value is identical to the 72-h WT mandated by KHRC. The IUC (25 pg/mL) was 8 times lower than the international HSL for urine (200 pg/mL). As risk management in urine sample testing and policy-making incorporates both scientific evidence and relevant contextual information [68], this HSL was likely set with consideration for the possibility of inadvertent positive exposure, such as feed contamination.

There is advocacy for the use of modeling approaches such as PBPK, population PK, and PK/PD, to allow for non-invasive investigations in veterinary clinical and research settings [69]. Our use of model-based meta-analysis offers a broad perspective into the PK and PD of DEX by integrating and summarizing existing data from diverse sources. This model sought to maximize the mechanistic nature as much as the available data allowed. A key strength of the developed mPBPK/PD model lies in its ability to simulate scenarios where experimental data may be lacking due to financial or ethical constraints. It also serves as a valuable tool to support prescribing veterinarians and regulatory agencies in estimating appropriate withdrawal times and optimizing dosing regimens for legitimate treatments without the need for invasive sampling. Regarding model translation across species, we have previously published relevant work on dexamethasone and other glucocorticoids [18, 41, 70] with considerations that such analyses were for teleological comparisons rather than human prediction.

The current meta-analysis has some limitations and of clarifications. All the datasets used for modeling were mean values either digitized (by necessity) or previously obtained by the authors. In some cases, they represented aggregated data that did not differentiate between sex, breed, or other significant factors, where considerable PK and PD variability might be expected [71]. In addition, most of the studies were performed under classic laboratory conditions rather than field conditions, without training and exercise that might have impact on drug disposition. Expectations for the time when plasma or urine concentrations decline to a proposed threshold should consider such variability by considering the 95% confidence intervals of both concentrations and times. Other studies reporting DEX PK following nebulized and topical administration, as well as plasma lactate responses, could not be incorporated into our model due to insufficient data quality and richness.

## Conclusions

This meta-analysis provided a comprehensive assessment of DEX PK/PD in horses utililizing all relevant data across doses and formulations. The developed mPBPK and PD models captured the central trends of the observations and demonstrated excellent predictive performance for measured data from individual horses. These models are robust and offer potential for predicting drug exposure and responses in both routine equine clinical practice and horseracing regulatory settings, and may be of interest to pharmaceutical scientists developing drugs for veterinary purposes. Similarly, our meta-analysis approach may be useful to the World Anti-Doping Agency in that most corticosteroids (except inhaled and topical) are banned in competitive human athletes and pharmacometric insights will be helpful in identifying washout conditions after legitimate therapeutic use [72].

## Supplementary Information

Below is the link to the electronic supplementary material.

Supplementary file1 (DOCX 345 KB)

## Data Availability

The authors declare that all the data utilized in this study are available in the figures in this article. Further datasets digitized and/or analyzed during the study are available from the corresponding author upon reasonable request.
