# Supplementary material for: Meta-Analysis and Physiologically-Based Modeling of the Pharmacokinetics and Pharmacodynamics of Dexamethasone in Horses
Source: Pharm Res. 2026 Jun 19;43(8):2733–50. doi: 10.1007/s11095-026-04120-5 (PMC13593757; doi:10.1007/s11095-026-04120-5)
Supplement: Supplementary file 1 [file 11095_2026_4120_MOESM1_ESM.docx]

**Supplemental Materials**

**Meta-Analysis and Physiologically-Based Modeling of the Pharmacokinetics and Pharmacodynamics of Dexamethasone in Horses**

Ruihong Yu^1^, Pierre-Louis Toutain^2^, Carl Ekstrand^3^, William J. Jusko^1*^

1. Department of Pharmaceutical Sciences, School of Pharmacy and Pharmaceutical Sciences, State University of New York at Buffalo, Buffalo, New York.

2. Department of Comparative Biomedical Sciences, The Royal Veterinary College, University of London, London, United Kingdom.

3. Department of Animal Biosciences, Swedish University of Agricultural Sciences, Uppsala, Sweden

Runing title: Pharmacokinetics and pharmacodynamics of dexamethasone in horses

*Corresponds to:

William J Jusko, Ph.D., Division of Pharmacokinetics, Pharmacodynamics, and Systems Pharmacology, Department of Pharmaceutical Sciences, School of Pharmacy and Pharmaceutical Sciences, State University of New York at Buffalo, 404 Pharmacy Building, Buffalo, NY 14214-8033. Telephone: 716-645-2855, Fax: 716-829-6569, E-mail: [wjjusko@buffalo.edu](mailto:wjjusko@buffalo.edu)

Table S1. Physiologic parameters utilized in the minimal physiologically based pharmacokinetic model.

| Parameter | Horse |
| --- | --- |
| Typical body weight (kg) | 550 |
| Blood and tissue volumes (*V_i_*, L) | |
| Blood (*V_b_*) | 38.90 |
| Liver (*V_l_*) | 7.15 |
| Remainder (*V_r_*) | 504 |
| Cardiac output and blood flow to tissues (*Q_i_*, L/h) | |
| Cardiac output (*Q_co_*) | 1964 |
| Liver (*Q_l_*) | 652 |
| Remainder (*Q_r_*) | 1312 |

Figure S1. Ruihong Yu and William J. Jusko. Aug 3, 2025.

**1. ADAPT code for the pharmacokinetic analysis of dexamethasone**

C**********************************************************************

C	ADAPT	*

C	Version 5	*

C**********************************************************************

C	MODEL - EXAMPLE	*

C	This file contains Fortran subroutines into which the user	*

C	must enter the relevant model equations and constants. *

C	Consult the User's Guide for details concerning the format for	*

C	entered equations and definition of symbols. *

C	1. Symbol- Parameter symbols and model constants	*

C	2. DiffEq- System differential equations	*

C	3. Output- System output equations	*

C	4. Varmod- Error variance model equations	*

C	5. Covmod- Covariate model equations (ITS,MLEM)	*

C	6. Popinit- Population parameter initial values (ITS,MLEM)	*

C	7. Prior - Parameter mean and covariance values (ID,NPD,STS) *

C	8. Sparam- Secondary parameters	*

C	9. Amat - System state matrix	*

C**********************************************************************

C######################################################################C

Subroutine SYMBOL

Implicit None

Include 'globals.inc'

Include 'model.inc'

C----------------------------------------------------------------------C

C	Enter as Indicated	C

C----c-----------------------------------------------------------------C

NDEqs	= 82 !Enter # of Diff. Eqs.

NSParam = 20 ! Enter # of System Parameters.

NVparam = 6 ! Enter # of Variance Parameters.

NSecPar = 0	! Enter # of Secondary Parameters.

NSecOut = 0 ! Enter # of Secondary Outputs (not used).i

Ieqsol = 1 ! Model type: 1 - DIFFEQ, 2 - AMAT, 3 - OUTPUT only.

Descr	= 'DEX mPBPK in horses'

C----------------------------------------------------------------------C

C	Enter Symbol for Each System Parameter (eg. Psym(1)='Kel')	C

C----c-----------------------------------------------------------------C

Psym(1)= 'Bmax1'

Psym(2)= 'kp2'

Psym(3)= 'Kd'

Psym(4)= 'CL10'

Psym(5)= 'Kfs'	! systemic Kf of ISO

Psym(6)= 'Kfp'	! systemic Kf of PHO

Psym(7)= 'CLr'

Psym(8)= 'Fs'	! IV F of ISO, conversion loss

Psym(9)= 'Fp'	! IV, IM F of PHO, conversion loss in system

Psym(10)= 'kas'	! IM, ka of ISO

Psym(11)= 'kal'	! IM, ka of Alcohol

Psym(12)= 'kao1'	! oral suspension

Psym(13)= 'kao2'	! oral powder

Psym(14)= 'Fp1'	! IA, F of PHO due to incomplete absoption

Psym(15)= 'Fml'	! IM, F of alcohol

Psym(16)= 'F1'	! oral suspension

Psym(17)= 'F2'	! oral powder

Psym(18)= 'kap'	! IM, ka of PHO

Psym(19)= 'Kfp1'	! IA, covnersion of PHO

Psym(20)= 'Kap1'	! IA, absoption rate constnat of PHO

C----------------------------------------------------------------------C

C	Enter Symbol for Each Variance Parameter {eg: PVsym(1)='Sigma'}	C

C----c-----------------------------------------------------------------C

PVsym(1)='sigma1'

PVsym(2)='intercept1'

PVsym(3)='sigma2'

PVsym(4)='intercept2'

PVsym(5)='sigma3'

PVsym(6)='intercept3'

C######################################################################C

Subroutine DIFFEQ(T,X,XP)

Implicit None

Include 'globals.inc'

Include 'model.inc'

Real*8 T,X(MaxNDE),XP(MaxNDE)

Real*8 Bmax1,Bmax2, CL10, Kd, Kfs, Kfp, CLr, Fs, Fp

Real*8 Rb_L, fup, Ka1, Kb, Kc,Vj,Fr2,Fr3,CLuf

Real*8 BW, Vp, V1, V2, Qco, Q1, Q2, Rb, MWd, MWs, MWp

Real*8 Kp1_a, Kp1_b, Kp1_c, Kp1_d, Kp1_e, Kp1_f, Kp1_g, Kp1_h

Real*8 Kp1_i, Kp1_j, Kp1_k, Kp1_l, Kp1_m, Kp1_n, Kp1_o, Kp1_p

Real*8 Kp1_q, Kp1_r, Kp1_s, Kp1_t, Kp1_u

Real*8 Kp2

Real*8 Kas, Kal, Fml,Kao1,kao2,F1,F2,ktr,kap,kap1,kfp1,Fp1

C----------------------------------------------------------------------C

C	Enter Differential Equations Below {e.g. XP(1) = -P(1)*X(1) }	C

C----c-----------------------------------------------------------------C

Bmax1=P(1)

Kp2=P(2)

Kd=P(3)

CL10=P(4)

Kfs=P(5)

Kfp=P(6)

CLr=P(7)

Fs=P(8)

Fp=P(9)

kas=P(10)

kal=P(11)

kao1=P(12)

kao2=P(13)

Fp1=P(14)

Fml=P(15)

F1=P(16)

F2=P(17)

kap=P(18)

Kfp1=P(19)

Kap1=P(20)

! Physiological parameters unit: V-mL, Q-mL/h, 1g =1mL

BW = 550000

Vp = 38896

V1 = 7150

V2 = 503954

Qco= 1963540

Q1 = 651550

Q2 =1311990

Rb =0.6945	! 0.664 male 0.725 female

MWd=392.46

MWs=492.54

MWp=516.41

C....... Model differential equations

Kp1_a= (-(X(3) - Kd -Bmax1) + ((X(3)- Kd

1	-Bmax1)**2+4*Kd*X(3)) **0.5)/(2*Kd)

Kp1_b= (-(X(6) - Kd -Bmax1) + ((X(6)- Kd

1	-Bmax1)**2+4*Kd*X(6)) **0.5)/(2*Kd)

Kp1_c= (-(X(10) - Kd -Bmax1) + ((X(10)- Kd

1	-Bmax1)**2+4*Kd*X(10)) **0.5)/(2*Kd)

Kp1_d= (-(X(14) - Kd -Bmax1) + ((X(14)- Kd

1	-Bmax1)**2+4*Kd*X(14)) **0.5)/(2*Kd)

Kp1_e= (-(X(18) - Kd -Bmax1) + ((X(18)- Kd

1	-Bmax1)**2+4*Kd*X(18)) **0.5)/(2*Kd)

Kp1_f= (-(X(21) - Kd -Bmax1) + ((X(21)- Kd

1	-Bmax1)**2+4*Kd*X(21)) **0.5)/(2*Kd)

Kp1_g= (-(X(24) - Kd -Bmax1) + ((X(24)- Kd

1	-Bmax1)**2+4*Kd*X(24)) **0.5)/(2*Kd)

Kp1_h= (-(X(28) - Kd -Bmax1) + ((X(28)- Kd

1	-Bmax1)**2+4*Kd*X(28)) **0.5)/(2*Kd)

Kp1_i= (-(X(32) - Kd -Bmax1) + ((X(32)- Kd

1	-Bmax1)**2+4*Kd*X(32)) **0.5)/(2*Kd)

Kp1_j= (-(X(36) - Kd -Bmax1) + ((X(36)- Kd

1	-Bmax1)**2+4*Kd*X(36)) **0.5)/(2*Kd)

Kp1_k= (-(X(39) - Kd -Bmax1) + ((X(39)- Kd

1	-Bmax1)**2+4*Kd*X(39)) **0.5)/(2*Kd)

Kp1_l= (-(X(42) - Kd -Bmax1) + ((X(42)- Kd

1	-Bmax1)**2+4*Kd*X(42)) **0.5)/(2*Kd)

Kp1_m= (-(X(47) - Kd -Bmax1) + ((X(47)- Kd

1	-Bmax1)**2+4*Kd*X(47)) **0.5)/(2*Kd)

Kp1_n= (-(X(51) - Kd -Bmax1) + ((X(51)- Kd

1	-Bmax1)**2+4*Kd*X(51)) **0.5)/(2*Kd)

Kp1_o= (-(X(55) - Kd -Bmax1) + ((X(55)- Kd

1	-Bmax1)**2+4*Kd*X(55)) **0.5)/(2*Kd)

Kp1_p= (-(X(60) - Kd -Bmax1) + ((X(60)- Kd

1	-Bmax1)**2+4*Kd*X(60)) **0.5)/(2*Kd)

Kp1_q= (-(X(65) - Kd -Bmax1) + ((X(65)- Kd

1	-Bmax1)**2+4*Kd*X(65)) **0.5)/(2*Kd)

Kp1_r= (-(X(69) - Kd -Bmax1) + ((X(69)- Kd

1	-Bmax1)**2+4*Kd*X(69)) **0.5)/(2*Kd)

Kp1_s= (-(X(74) - Kd -Bmax1) + ((X(74)- Kd

1	-Bmax1)**2+4*Kd*X(74)) **0.5)/(2*Kd)

Kp1_t= (-(X(81) - Kd -Bmax1) + ((X(81)- Kd

1	-Bmax1)**2+4*Kd*X(81)) **0.5)/(2*Kd)

Kp1_u= (-(X(76) - Kd -Bmax1) + ((X(76)- Kd

1	-Bmax1)**2+4*Kd*X(76)) **0.5)/(2*Kd)

C........12 IV doses...........................................................................

c	Dose 1	Ref1-DEX-ISO 0.05 mg/kg 556.67 kg

c	1. Formation-DEX-ISO

XP(1) = -Kfs*X(1)

c	2. plasma-DEX

XP(2) = (Kfs*Fs*X(1)*MWd/MWs+Q1*X(3)/Kp1_a

1	+ Q2*X(4)/Kp2 - Qco *X(2)- CLr*X(2))/Vp/Rb

c	3. liver-DEX

XP(3) = (Q1*(X(2)- X(3)/Kp1_a))/V1-(CL10*X(3)/Kp1_a)/V1

c	4. remainder-DEX

XP(4) = (Q2*(X(2)-X(4)/Kp2))/V2

c	Dose 2	Ref1-DEX-ALC 0.05 mg/kg 556.67 kg

XP(5) = (Q1*X(6)/Kp1_b

1	+ Q2*X(7)/Kp2 - Qco *X(5)- CLr*X(5))/Vp/Rb

XP(6) = (Q1*(X(5)- X(6)/Kp1_b))/V1-(CL10*X(6)/Kp1_b)/V1

XP(7) = (Q2*(X(5)-X(7)/Kp2))/V2

c	Dose 3	Ref45-DEX-PHO low 507.92 kg

XP(8) = R(1) - Kfp*X(8)	! Formation-DEX-PHO

XP(9) = (Kfp*Fp*X(8)*MWd/MWp+Q1*X(10)/Kp1_c

1	+ Q2*X(11)/Kp2 - Qco *X(9)- CLr*X(9))/Vp/Rb

XP(10) = (Q1*(X(9)- X(10)/Kp1_c))/V1-(CL10*X(10)/Kp1_c)/V1

XP(11) = (Q2*(X(9)-X(11)/Kp2))/V2

c	Dose 4	Ref45-DEX-PHO med

XP(12) = R(2) -Kfp*X(12)

XP(13) = (Kfp*Fp*X(12)*MWd/MWp+Q1*X(14)/Kp1_d

1	+ Q2*X(15)/Kp2 - Qco *X(13)- CLr*X(13))/Vp/Rb

XP(14) = (Q1*(X(13)- X(14)/Kp1_d))/V1-(CL10*X(14)/Kp1_d)/V1

XP(15) = (Q2*(X(13)-X(15)/Kp2))/V2

c	Dose 5	Ref45-DEX-PHO high

XP(16) = R(3) - Kfp*X(16)

XP(17) = (Kfp*Fp*X(16)*MWd/MWp+Q1*X(18)/Kp1_e

1	+ Q2*X(19)/Kp2 - Qco *X(17)- CLr*X(17))/Vp/Rb

XP(18) = (Q1*(X(17)- X(18)/Kp1_e))/V1-(CL10*X(18)/Kp1_e)/V1

XP(19) = (Q2*(X(17)-X(19)/Kp2))/V2

c	Dose 6	Ref6-DEX-ALC 0.008 mg/kg 522.63 kg

XP(20) = (Q1*X(21)/Kp1_f

1	+ Q2*X(22)/Kp2 - Qco *X(20)- CLr*X(20))/Vp/Rb

XP(21) = (Q1*(X(20)- X(21)/Kp1_f))/V1-(CL10*X(21)/Kp1_f)/V1

XP(22) = (Q2*(X(20)-X(22)/Kp2))/V2

c	Dose 7	Ref8-DEX 0.05 mg/kg 514.5 kg

XP(23) = (Q1*X(24)/Kp1_g

1	+ Q2*X(25)/Kp2 - Qco *X(23)- CLr*X(23))/Vp/Rb

XP(24) = (Q1*(X(23)- X(24)/Kp1_g))/V1-(CL10*X(24)/Kp1_g)/V1

XP(25) = (Q2*(X(23)-X(25)/Kp2))/V2

c	Dose 8	Ref9-DEX-PHO 0.05 mg/kg 523.5 kg

XP(26) = -Kfp*X(26)

XP(27) = (Kfp*Fp*X(26)*MWd/MWp+Q1*X(28)/Kp1_h

1	+ Q2*X(29)/Kp2 - Qco *X(27)- CLr*X(27))/Vp/Rb

XP(28) = (Q1*(X(27)- X(28)/Kp1_h))/V1-(CL10*X(28)/Kp1_h)/V1

XP(29) = (Q2*(X(27)-X(29)/Kp2))/V2

c	Dose 9	Ref10-DEX-PHO 0.075 mg/kg 534 kg

XP(30) = -Kfp*X(30)

XP(31) = (Kfp*Fp*X(30)*MWd/MWp+Q1*X(32)/Kp1_i

1	+ Q2*X(33)/Kp2 - Qco *X(31)- CLr*X(31))/Vp/Rb

XP(32) = (Q1*(X(31)- X(32)/Kp1_i))/V1-(CL10*X(32)/Kp1_i)/V1

XP(33) = (Q2*(X(31)-X(33)/Kp2))/V2

c	Dose 10	Ref11-DEX-PHO 0.0096 mg/kg 521 kg

XP(34) = -Kfp*X(34)

XP(35) = (Kfp*Fp*X(34)*MWd/MWp+Q1*X(36)/Kp1_j

1	+ Q2*X(37)/Kp2 - Qco *X(35)- CLr*X(35))/Vp/Rb

XP(36) = (Q1*(X(35)- X(36)/Kp1_j))/V1-(CL10*X(36)/Kp1_j)/V1

XP(37) = (Q2*(X(35)-X(37)/Kp2))/V2

c	Dose 11	Ref12-DEX 0.0225 mg/kg 443.7 kg

XP(38) = (Q1*X(39)/Kp1_k

1	+ Q2*X(40)/Kp2 - Qco *X(38)- CLr*X(38))/Vp/Rb

XP(39) = (Q1*(X(38)- X(39)/Kp1_k))/V1-(CL10*X(39)/Kp1_k)/V1

XP(40) = (Q2*(X(38)-X(40)/Kp2))/V2

c	Dose 12	Ref16-DEX 0.05 mg/kg 507.5 kg

XP(41) = (Q1*X(42)/Kp1_l

1	+ Q2*X(43)/Kp2 - Qco *X(41)-CLr*X(41))/Vp/Rb

XP(42) = (Q1*(X(41)- X(42)/Kp1_l))/V1-(CL10*X(42)/Kp1_l)/V1

XP(43) = (Q2*(X(41)-X(43)/Kp2))/V2

c	Dose 13	Ref3-IM Ref1-DEX_ISO 0.0239 mg/kg 474.17 kg

XP(44) = -Kas*X(44)

XP(45) = Kas*X(44)-Kfs*X(45)

XP(46) = (Kfs*Fs*X(45)*MWd/MWs+Q1*X(47)/Kp1_m

1	+ Q2*X(48)/Kp2 - Qco *X(46)- CLr*X(46))/Vp/Rb

XP(47) = (Q1*(X(46)- X(47)/Kp1_m))/V1-(CL10*X(47)/Kp1_m)/V1

XP(48) = (Q2*(X(46)-X(48)/Kp2))/V2

c	Dose 14	Ref1-IM Ref1-DEX_ALC 0.05 mg/kg 556.67 kg

XP(49) = -Kal*X(49)

XP(50) = (Kal*Fml*X(49)+Q1*X(51)/Kp1_n

1	+ Q2*X(52)/Kp2- Qco *X(50)- CLr*X(50))/Vp/Rb

XP(51) = (Q1*(X(50)- X(51)/Kp1_n))/V1-(CL10*X(51)/Kp1_n)/V1

XP(52) = (Q2*(X(50)-X(52)/Kp2))/V2

c	Dose 15	Ref9-oral DEX suspension 0.05 mg/kg

XP(53) = -Kao1*X(53)

XP(54) = (Q1*X(55)/Kp1_o

1	+ Q2*X(56)/Kp2- Qco *X(54)- CLr*X(54))/Vp/Rb

XP(55) = (Kao1*F1*X(53)+Q1*(X(54)- X(55)/Kp1_o))/V1

1	-(CL10*X(55)/Kp1_o)/V1

XP(56) = (Q2*(X(54)-X(56)/Kp2))/V2

c	Dose 16	Ref10-oral DEX tablet 0.05576 mg/kg

XP(57) = -Kao2*X(57)

XP(59) = (Q1*X(60)/Kp1_p

1	+ Q2*X(61)/Kp2 - Qco *X(59)- CLr*X(59))/Vp/Rb

XP(60) = (Kao2*F2*X(57)+Q1*(X(59)- X(60)/Kp1_p))/V1

1	-(CL10*X(60)/Kp1_p)/V1

XP(61) = (Q2*(X(59)-X(61)/Kp2))/V2

c	Dose 17	Ref12-oral DEX powder 0.02254 mg/kg multiple doses

XP(62) = -Kao2*X(62)

XP(64) = (Q1*X(65)/Kp1_q

1	+ Q2*X(66)/Kp2 - Qco *X(64)- CLr*X(64))/Vp/Rb

XP(65) = (Kao2*F2*X(62)+Q1*(X(64)- X(65)/Kp1_q))/V1

1	-(CL10*X(65)/Kp1_q)/V1

XP(66) = (Q2*(X(64)-X(66)/Kp2))/V2

c	Dose 18	Ref16-oral DEX solution 0.05 mg/kg

XP(67) = -Kao1*X(67)

XP(68) = (Q1*X(69)/Kp1_r

1	+ Q2*X(70)/Kp2- Qco *X(68)- CLr*X(68))/Vp/Rb

XP(69) = (Kao1*F1*X(67)+Q1*(X(68)- X(69)/Kp1_r))/V1

1	-(CL10*X(69)/Kp1_r)/V1

XP(70) = (Q2*(X(68)-X(70)/Kp2))/V2

c	Dose 19	Ref16-oral DEX powder 0.05 mg/kg

XP(71) = -Kao2*X(71)

XP(73) = (Q1*X(74)/Kp1_s

1	+ Q2*X(75)/Kp2 - Qco *X(73)- CLr*X(73))/Vp/Rb

XP(74) = (Kao2*F2*X(71)+Q1*(X(73)- X(74)/Kp1_s))/V1

1	-(CL10*X(74)/Kp1_s)/V1

XP(75) = (Q2*(X(73)-X(75)/Kp2))/V2

c	Dose 20	Ref9-IM Ref1-DEX_PHO 0.05 mg/kg

XP(78) = -Kap*X(78)

XP(79) = Kap*X(78)-Kfp*X(79)

XP(80) = (Kfp*Fp*X(79)*MWd/MWp+Q1*X(81)/Kp1_t

1	+ Q2*X(82)/Kp2 - Qco *X(80)- CLr*X(80))/Vp/Rb

XP(81) = (Q1*(X(80)- X(81)/Kp1_t))/V1-(CL10*X(81)/Kp1_t)/V1

XP(82) = (Q2*(X(80)-X(82)/Kp2))/V2

c	Dose 21	Ref9-IA Ref1-DEX_PHO 0.05 mg/kg

XP(58) = -Kfp1*X(58) ! prodrug in depot

XP(63) = Kfp1*X(58)*MWd/MWp-Kap1*X(63) ! DEX in joint

XP(72) = (Kap1*Fp1*X(63)+Q1*X(76)/Kp1_u

1	+ Q2*X(77)/Kp2 - Qco *X(72)- CLr*X(72))/Vp/Rb

XP(76) = (Q1*(X(72)- X(76)/Kp1_u))/V1-(CL10*X(76)/Kp1_u)/V1

XP(77) = (Q2*(X(72)-X(77)/Kp2))/V2

C----------------------------------------------------------------------C

Return

End

C####################################################################C

Subroutine OUTPUT(Y,T,X)

Implicit None

Include 'globals.inc'

Include 'model.inc'

Real*8 Y(MaxNOE),T,X(MaxNDE)

Integer DelayPar

Real*8 CLr

C----------------------------------------------------------------------C

C	Enter Output Equations Below	{e.g. Y(1) = X(1)/P(2) }	C

C----c-----------------------------------------------------------------C

CLr= P(7)

! IV plasma

Y(1) = X(2)

Y(2) = X(5)

Y(3) = X(9)

Y(4) = X(13)

Y(5) = X(17)

Y(6) = X(20)

Y(7) = X(23)

Y(8) = X(27)

Y(9) = X(31)

Y(10) = X(35)

Y(11) = X(38)

Y(12) = X(41)

! IV urine

Y(13) = CLr*X(20)/494

! IM

Y(14) = X(46)

Y(15) = X(50)

! oral

Y(16) = X(54)

Y(17) = X(59)

Y(18) = X(64)

Y(19) = X(68)

Y(20) = X(73)

! IM Ref 9

Y(21) = X(80)

! IA

Y(22) = X(72)

! IM urine

Y(23) = CLr*X(46)/494

C----------------------------------------------------------------------C

Return

End

C######################################################################C

Subroutine VARMOD(V,T,X,Y)

Implicit None

Include 'globals.inc'

Include 'model.inc'

Real*8 V(MaxNOE),T,X(MaxNDE),Y(MaxNOE)

C----------------------------------------------------------------------C

C	Enter Variance Model Equations Below	C

C	{e.g. V(1) = (PV(1) + PV(2)*Y(1))**2 }	C

C----c-----------------------------------------------------------------C

V(1:12) = (PV(2) + PV(1)*Y(1:12))**2

V(13) = (PV(4) + PV(3)*Y(13))**2

V(14:15) = (PV(6) + PV(5)*Y(14:15))**2

V(16:20) = (PV(2) + PV(1)*Y(16:20))**2

V(21:22) = (PV(6) + PV(5)*Y(21:22))**2

V(23) = (PV(4) + PV(3)*Y(23))**2

C----------------------------------------------------------------------C

C----------------------------------------------------------------------C

Return

End

C######################################################################C

**2. ADAPT code for the pharmacodynamic analysis of dexamethasone -Cortisol**

C**********************************************************************

C	ADAPT	*

C	Version 5	*

C**********************************************************************

C	MODEL - EXAMPLE	*

C	This file contains Fortran subroutines into which the user	*

C	must enter the relevant model equations and constants. *

C	Consult the User's Guide for details concerning the format for	*

C	entered equations and definition of symbols. *

C	1. Symbol- Parameter symbols and model constants	*

C	2. DiffEq- System differential equations	*

C	3. Output- System output equations	*

C	4. Varmod- Error variance model equations	*

C	5. Covmod- Covariate model equations (ITS,MLEM)	*

C	6. Popinit- Population parameter initial values (ITS,MLEM)	*

C	7. Prior - Parameter mean and covariance values (ID,NPD,STS) *

C	8. Sparam- Secondary parameters	*

C	9. Amat - System state matrix	*

C**********************************************************************

C######################################################################C

Subroutine SYMBOL

Implicit None

Include 'globals.inc'

Include 'model.inc'

C----------------------------------------------------------------------C

C	Enter as Indicated	C

C----c-----------------------------------------------------------------C

NDEqs	= 61 !Enter # of Diff. Eqs.

NSParam = 8 ! Enter # of System Parameters.

NVparam = 12 ! Enter # of Variance Parameters.

NSecPar = 6	! Enter # of Secondary Parameters.

NSecOut = 0 ! Enter # of Secondary Outputs (not used).i

Ieqsol = 1 ! Model type: 1 - DIFFEQ, 2 - AMAT, 3 - OUTPUT only.

Descr	= 'DEX cortisol in horses'

C----------------------------------------------------------------------C

C	Enter Symbol for Each System Parameter (eg. Psym(1)='Kel')	C

C----c-----------------------------------------------------------------C

Psym(1)= 'IC50'

Psym(2)= 'Tau'

Psym(3)= 'kd1'

Psym(4)= 'Rmax1'

Psym(5)= 'Rmax5'

Psym(6)= 'Rmax6'

Psym(7)= 'Imax'

Psym(8)= 'Gama'

C----------------------------------------------------------------------C

C	Enter Symbol for Each Variance Parameter {eg: PVsym(1)='Sigma'}	C

C----c-----------------------------------------------------------------C

PVsym(1)='sigma1'

PVsym(2)='intercept1'

PVsym(3)='sigma2'

PVsym(4)='intercept2'

PVsym(5)='sigma3'

PVsym(6)='intercept3'

PVsym(7)='sigma4'

PVsym(8)='intercept4'

PVsym(9)='sigma5'

PVsym(10)='intercept5'

PVsym(11)='sigma6'

PVsym(12)='intercept6'

C----------------------------------------------------------------------C

C	Enter Symbol for Each Secondary Parameter {eg: PSsym(1)='CLt'}	C

C----c-----------------------------------------------------------------C

PSsym(1)='Rm1'

PSsym(2)='Rm2'

PSsym(3)='Rm3'

PSsym(4)='Rm4'

PSsym(5)='Rm5'

PSsym(6)='Rm6'

C----------------------------------------------------------------------C

Return

End

C######################################################################C

Subroutine DIFFEQ(T,X,XP)

Implicit None

Include 'globals.inc'

Include 'model.inc'

Real*8 T,X(MaxNDE),XP(MaxNDE)

Real*8 Bmax1,CL10, Kd, Kfs, Kfp, CLr, Fs, Fp

Real*8 Rb_L, fup, Ka1, Kb, Kc,Vj,Fr2,Fr3,CLuf

Real*8 BW, Vp, V1, V2, Qco, Q1, Q2, Rb, MWd, MWs, MWp

Real*8 Kp1_a, Kp1_b, Kp1_c, Kp1_d, Kp1_e, Kp1_f, Kp1_g, Kp1_h

Real*8 Kp1_i, Kp1_j, Kp1_k

Real*8 Kp2

Real*8 Kas, Kal, Fml,Kao1,kao2,F1,F2,ktr,kap,kap1,kfp1,Fp1

Real*8 IC50, Rmax1, Ks1,Rmax2, Ks2,Rmax3, Ks3,Rmax4, Ks4

Real*8 Rmax5, Ks5,Rmax6, Ks6, kd1

Real*8 tp1, tp2, tp3, tp4,Ra,Tau, Imax, Gama,Ra1

Real*8 Rm1, Rm2

C----------------------------------------------------------------------C

C	Enter Differential Equations Below {e.g. XP(1) = -P(1)*X(1) }	C

C----c-----------------------------------------------------------------C

IC50=P(1)

Tau=P(2)

kd1=P(3)

Rmax1=P(4)

Rmax5=P(5)

Rmax6=P(6)

Imax=P(7)

Gama=P(8)

Rmax2=0

Rmax3=0

Rmax4=0

! Physiological parameters	! unit: V-mL, Q-mL/h, 1g =1mL

BW = 550000

Vp = 38896

V1 = 7150

V2 = 503954

Qco=1963540

Q1 = 651550

Q2 =1311990

Rb =0.6945	! 0.664 male 0.725 female

fup = 0.175

MWd=392.46

MWs=492.54

MWp=516.41

Bmax1=140.1

Kp2=1.09

Kd=0.269

CL10=260500

Kfs=20.59

Kfp=16.67

CLr=3131

Fs=0.6951

Fp=1

kas=0.01052

kal=0.1312

kao1=0.5291

kao2=0.4212

Fp1=0.5642

Fml=1

F1=0.6622

F2=0.5210

kap=2.949

Kfp1=30

Kap1=4.126

C....... Model differential equations

C........13 doses PK.........................................................................

Kp1_a= (-(X(3) - Kd -Bmax1) + ((X(3)- Kd

1	-Bmax1)**2+4*Kd*X(3)) **0.5)/(2*Kd)

Kp1_b= (-(X(6) - Kd -Bmax1) + ((X(6)- Kd

1	-Bmax1)**2+4*Kd*X(6)) **0.5)/(2*Kd)

Kp1_c= (-(X(10) - Kd -Bmax1) + ((X(10)- Kd

1	-Bmax1)**2+4*Kd*X(10)) **0.5)/(2*Kd)

Kp1_d= (-(X(15) - Kd -Bmax1) + ((X(15)- Kd

1	-Bmax1)**2+4*Kd*X(15)) **0.5)/(2*Kd)

Kp1_e= (-(X(19) - Kd -Bmax1) + ((X(19)- Kd

1	-Bmax1)**2+4*Kd*X(19)) **0.5)/(2*Kd)

Kp1_f= (-(X(23) - Kd -Bmax1) + ((X(23)- Kd

1	-Bmax1)**2+4*Kd*X(23)) **0.5)/(2*Kd)

Kp1_g= (-(X(27) - Kd -Bmax1) + ((X(27)- Kd

1	-Bmax1)**2+4*Kd*X(27)) **0.5)/(2*Kd)

Kp1_h= (-(X(31) - Kd -Bmax1) + ((X(31)- Kd

1	-Bmax1)**2+4*Kd*X(31)) **0.5)/(2*Kd)

Kp1_i= (-(X(36) - Kd -Bmax1) + ((X(36)- Kd

1	-Bmax1)**2+4*Kd*X(36)) **0.5)/(2*Kd)

Kp1_j= (-(X(40) - Kd -Bmax1) + ((X(40)- Kd

1	-Bmax1)**2+4*Kd*X(40)) **0.5)/(2*Kd)

Kp1_k= (-(X(44) - Kd -Bmax1) + ((X(44)- Kd

1	-Bmax1)**2+4*Kd*X(44)) **0.5)/(2*Kd)

c	Dose 1	1984Ref1-IV DEX-ISO DEX-0.05 mg/kg 556.67 kg 8AM

c	1. Formation-DEX-ISO

XP(1) = -Kfs*X(1)

c	2. plasma-DEX

XP(2) = (Kfs*Fs*X(1)*MWd/MWs+Q1*X(3)/Kp1_a

1	+ Q2*X(4)/Kp2 - Qco *X(2)- CLr*X(2))/Vp/Rb

c	3. liver-DEX

XP(3) = (Q1*(X(2)- X(3)/Kp1_a))/V1-(CL10*X(3)/Kp1_a)/V1

c	4. remainder-DEX

XP(4) = (Q2*(X(2)-X(4)/Kp2))/V2

c	Dose 2	1984Ref1-IV-DEX-ALC DEX-0.05 mg/kg 556.67 kg 8AM

c	Dose 8 2005Ref8-IV-DEX DEX-0.05 mg/kg	7AM

c	Dose 11 2010Ref16-IV-DEX DEX-0.05 mg/kg-feed	8AM

XP(5) = (Q1*X(6)/Kp1_b

1	+ Q2*X(7)/Kp2 - Qco *X(5)- CLr*X(5))/Vp/Rb

XP(6) = (Q1*(X(5)- X(6)/Kp1_b))/V1-(CL10*X(6)/Kp1_b)/V1

XP(7) = (Q2*(X(5)-X(7)/Kp2))/V2

c	Dose 3	1984Ref1-IM-DEX_ALC DEX-0.05 mg/kg 556.67 kg 8AM

XP(8) = -Kal*X(8)

XP(9) = (Kal*Fml*X(8)+Q1*X(10)/Kp1_c

1	+ Q2*X(11)/Kp2- Qco *X(9)- CLr*X(9))/Vp/Rb

XP(10) = (Q1*(X(9)- X(10)/Kp1_c))/V1-(CL10*X(10)/Kp1_c)/V1

XP(11) = (Q2*(X(9)-X(11)/Kp2))/V2

c	Dose 4	2014 Ref3-IM-DEX_ISO-0.03 mg/kg 9AM

XP(12) = -Kas*X(12)

XP(13) = Kas*X(12)-Kfs*X(13)

XP(14) = (Kfs*Fs*X(13)*MWd/MWs+Q1*X(15)/Kp1_d

1	+ Q2*X(16)/Kp2 - Qco *X(14)- CLr*X(14))/Vp/Rb

XP(15) = (Q1*(X(14)- X(15)/Kp1_d))/V1-(CL10*X(15)/Kp1_d)/V1

XP(16) = (Q2*(X(14)-X(16)/Kp2))/V2

c	Dose 5	2015Ref45-IV-DEX-PHO low DEX-0.1ug/kg+0.07ug/kg/h 1.556 PM

XP(17) = R(1) - Kfp*X(17)

XP(18) = (Kfp*Fp*X(17)*MWd/MWp+Q1*X(19)/Kp1_e

1	+ Q2*X(20)/Kp2 - Qco *X(18)- CLr*X(18))/Vp/Rb

XP(19) = (Q1*(X(18)- X(19)/Kp1_e))/V1-(CL10*X(19)/Kp1_e)/V1

XP(20) = (Q2*(X(18)-X(20)/Kp2))/V2

c	Dose 6	2015Ref45-IV-DEX-PHO med

XP(21) = R(2) -Kfp*X(21)

XP(22) = (Kfp*Fp*X(21)*MWd/MWp+Q1*X(23)/Kp1_f

1	+ Q2*X(24)/Kp2 - Qco *X(22)- CLr*X(22))/Vp/Rb

XP(23) = (Q1*(X(22)- X(23)/Kp1_f))/V1-(CL10*X(23)/Kp1_f)/V1

XP(24) = (Q2*(X(22)-X(24)/Kp2))/V2

c	Dose 7	2015Ref45-IV-DEX-PHO high

XP(25) = R(3) - Kfp*X(25)

XP(26) = (Kfp*Fp*X(25)*MWd/MWp+Q1*X(27)/Kp1_g

1	+ Q2*X(28)/Kp2 - Qco *X(26)- CLr*X(26))/Vp/Rb

XP(27) = (Q1*(X(26)- X(27)/Kp1_g))/V1-(CL10*X(27)/Kp1_g)/V1

XP(28) = (Q2*(X(26)-X(28)/Kp2))/V2

c	Dose 9	2005Ref9-IV-DEX_PHO DEX-0.05 mg/kg 8AM

XP(29) = -Kfp*X(29)

XP(30) = (Kfp*Fp*X(29)*MWd/MWp+Q1*X(31)/Kp1_h

1	+ Q2*X(32)/Kp2 - Qco *X(30)- CLr*X(30))/Vp/Rb

XP(31) = (Q1*(X(30)- X(31)/Kp1_h))/V1-(CL10*X(31)/Kp1_h)/V1

XP(32) = (Q2*(X(30)-X(32)/Kp2))/V2

c	Dose 10	2005Ref9-IA-DEX_PHO DEX-0.05 mg/kg 8AM

XP(33) = -Kfp1*X(33) ! prodrug in depot

XP(34) = Kfp1*X(33)*MWd/MWp-Kap1*X(34) ! DEX in joint

XP(35) = (Kap1*Fp1*X(34)+Q1*X(36)/Kp1_i

1	+ Q2*X(37)/Kp2 - Qco *X(35)- CLr*X(35))/Vp/Rb

XP(36) = (Q1*(X(35)- X(36)/Kp1_i))/V1-(CL10*X(36)/Kp1_i)/V1

XP(37) = (Q2*(X(35)-X(37)/Kp2))/V2

c	Dose 12	2010Ref16-oral DEX suspension DEX-0.05 mg/kg-feed 8AM

XP(38) = -Kao1*X(38)

XP(39) = (Q1*X(40)/Kp1_j

1	+ Q2*X(41)/Kp2 - Qco *X(39)- CLr*X(39))/Vp/Rb

XP(40) = (Kao1*F1*X(38)+Q1*(X(39)- X(40)/Kp1_j))/V1

1	-(CL10*X(40)/Kp1_j)/V1

XP(41) = (Q2*(X(39)-X(41)/Kp2))/V2

c	Dose 13	2010Ref16-oral DEX powder DEX-0.05 mg/kg-feed 8AM

XP(42) = -Kao2*X(42)

XP(43) = (Q1*X(44)/Kp1_k

1	+ Q2*X(45)/Kp2 - Qco *X(43)- CLr*X(43))/Vp/Rb

XP(44) = (Kao2*F2*X(42)+Q1*(X(43)- X(44)/Kp1_k))/V1

1	-(CL10*X(44)/Kp1_k)/V1

XP(45) = (Q2*(X(43)-X(45)/Kp2))/V2

C----------------------------------------------------------------------C

tp1 = 0.94

tp2 = 23.94

tp3 = 22.94

tp4= 1.94

Ra = 15.23	! mean

Ra1 = 18.62	! ref 4 and 16

Rm1 = 47.89

Rm2 = 93.42

C----baseline

C---Ref 1 8AM

ks1 = Kd1*Rm1 + Kd1*Ra*cos(2*pi/24*(t-tp1))

1	- 2*pi/24*Ra*sin(2*pi/24*(t-tp1))

C---Ref 3	9AM

ks2 = Kd1*Rm2 + Kd1*Ra*cos(2*pi/24*(t-tp2))

1	- 2*pi/24*Ra*sin(2*pi/24*(t-tp2))

C---Ref 4&5 10 AM

ks3= Kd1*Rm1 + Kd1*Ra1*cos(2*pi/24*(t-tp3))

1	- 2*pi/24*Ra1*sin(2*pi/24*(t-tp3))

C---Ref 8	7AM

ks4 = Kd1*Rm1 + Kd1*Ra*cos(2*pi/24*(t-tp4))

1	- 2*pi/24*Ra*sin(2*pi/24*(t-tp4))

C---Ref 9	8AM

ks5 = Kd1*Rm1+ Kd1*Ra*cos(2*pi/24*(t-tp1))

1	- 2*pi/24*Ra*sin(2*pi/24*(t-tp1))

C---Ref 16 8AM

ks6= Kd1*Rm2 + Kd1*Ra1*cos(2*pi/24*(t-tp1))

1	- 2*pi/24*Ra1*sin(2*pi/24*(t-tp1))

C---Ref 3

XP(46) = ks2 - kd1*X(46)

C---Ref 4&5

XP(47) = ks3 - kd1*X(47)

C---Ref 16

XP(48) = ks6 - kd1*X(48)

C----------------------------------------------------------------------C

C---PD： cortisol response

if	(t .lt. tau)	then

C---!Ref 1

XP(49) = Rmax1/tau + ks1*(1-(1*X(2))**Gama*Imax/

1	((1*X(2))**Gama+IC50**Gama))- kd1*X(49)

XP(50) = Rmax1/tau + ks1*(1-(1*X(5))**Gama*Imax/

1	((1*X(5))**Gama+IC50**Gama))- kd1*X(50)

XP(51) = Rmax1/tau + ks1*(1-(1*X(9))**Gama*Imax/

1	((1*X(9))**Gama+IC50**Gama))- kd1*X(51)

C---!Ref 3

XP(52) = Rmax2/tau + ks2*(1-(1*X(14))**Gama*Imax/

1	((1*X(14))**Gama+IC50**Gama))- kd1*X(52)

C---!Ref 4&5

XP(53) = Rmax3/tau + ks3*(1-(1*X(18))**Gama*Imax/

1	((1*X(18))**Gama+IC50**Gama))- kd1*X(53)

XP(54) = Rmax3/tau + ks3*(1-(1*X(22))**Gama*Imax/

1	((1*X(22))**Gama+IC50**Gama))- kd1*X(54)

XP(55) = Rmax3/tau + ks3*(1-(1*X(26))**Gama*Imax/

1	((1*X(26))**Gama+IC50**Gama))- kd1*X(55)

C---!Ref 8

XP(56) = Rmax4/tau + ks4*(1-(1*X(5))**Gama*Imax/

1	((1*X(5))**Gama+IC50**Gama))- kd1*X(56)

C---!Ref 9

XP(57) = Rmax5/tau + ks5*(1-(1*X(30))**Gama*Imax/

1	((1*X(30))**Gama+IC50**Gama))- kd1*X(57)

XP(58) = Rmax5/tau + ks5*(1-(1*X(35))**Gama*Imax/

1	((1*X(35))**Gama+IC50**Gama))- kd1*X(58)

C---!Ref 16

XP(59) = Rmax6/tau + ks6*(1-(1*X(5))**Gama*Imax/

1	((1*X(5))**Gama+IC50**Gama))- kd1*X(59)

XP(60) = Rmax6/tau + ks6*(1-(1*X(39))**Gama*Imax/

1	((1*X(39))**Gama+IC50**Gama))- kd1*X(60)

XP(61) = Rmax6/tau + ks6*(1-(1*X(43))**Gama*Imax/

1	((1*X(43))**Gama+IC50**Gama))- kd1*X(61)

else

XP(49) = ks1*(1-(1*X(2))**Gama*Imax/

1	((1*X(2))**Gama+IC50**Gama))- kd1*X(49)

XP(50) = ks1*(1-(1*X(5))**Gama*Imax/

1	((1*X(5))**Gama+IC50**Gama))- kd1*X(50)

XP(51) = ks1*(1-(1*X(9))**Gama*Imax/

1	((1*X(9))**Gama+IC50**Gama))- kd1*X(51)

XP(52) = ks2*(1-(1*X(14))**Gama*Imax/

1	((1*X(14))**Gama+IC50**Gama))- kd1*X(52)

XP(53) = ks3*(1-(1*X(18))**Gama*Imax/

1	((1*X(18))**Gama+IC50**Gama))- kd1*X(53)

XP(54) = ks3*(1-(1*X(22))**Gama*Imax/

1	((1*X(22))**Gama+IC50**Gama))- kd1*X(54)

XP(55) = ks3*(1-(1*X(26))**Gama*Imax/

1	((1*X(26))**Gama+IC50**Gama))- kd1*X(55)

XP(56) = ks4*(1-(1*X(5))**Gama*Imax/

1	((1*X(5))**Gama+IC50**Gama))- kd1*X(56)

XP(57) = ks5*(1-(1*X(30))**Gama*Imax/

1	((1*X(30))**Gama+IC50**Gama))- kd1*X(57)

XP(58) = ks5*(1-(1*X(35))**Gama*Imax/

1	((1*X(35))**Gama+IC50**Gama))- kd1*X(58)

XP(59) = ks6*(1-(1*X(5))**Gama*Imax/

1	((1*X(5))**Gama+IC50**Gama))- kd1*X(59)

XP(60) = ks6*(1-(1*X(39))**Gama*Imax/

1	((1*X(39))**Gama+IC50**Gama))- kd1*X(60)

XP(61) = ks6*(1-(1*X(43))**Gama*Imax/

1	((1*X(43))**Gama+IC50**Gama))- kd1*X(61)

endif

Return

End

C####################################################################C

Subroutine OUTPUT(Y,T,X)

Implicit None

Include 'globals.inc'

Include 'model.inc'

Real*8 Y(MaxNOE),T,X(MaxNDE)

Integer DelayPar

Real*8 CLr

C----------------------------------------------------------------------C

C	Enter Output Equations Below	{e.g. Y(1) = X(1)/P(2) }	C

C----c-----------------------------------------------------------------C

Y(1) = X(46)

Y(2) = X(47)

Y(3) = X(48)

Y(4) = X(49)

Y(5) = X(50)

Y(6) = X(51)

Y(7) = X(52)

Y(8) = X(53)

Y(9) = X(54)

Y(10) = X(55)

Y(11) = X(56)

Y(12) = X(57)

Y(13) = X(58)

Y(14) = X(59)

Y(15) = X(60)

Y(16) = X(61)

C----------------------------------------------------------------------C

Return

End

C######################################################################C

Subroutine VARMOD(V,T,X,Y)

Implicit None

Include 'globals.inc'

Include 'model.inc'

Real*8 V(MaxNOE),T,X(MaxNDE),Y(MaxNOE)

C----------------------------------------------------------------------C

C	Enter Variance Model Equations Below	C

C	{e.g. V(1) = (PV(1) + PV(2)*Y(1))**2 }	C

C----c-----------------------------------------------------------------C

V(1) = (PV(4) + PV(3)*Y(1))**2

V(2) = (PV(6) + PV(5)*Y(2))**2

V(3) = (PV(12) + PV(11)*Y(3))**2

V(4:6) = (PV(2) + PV(1)*Y(4:6))**2

V(7) = (PV(4) + PV(3)*Y(7))**2

V(8:10) = (PV(6) + PV(5)*Y(8:10))**2

V(11) = (PV(8) + PV(7)*Y(11))**2

V(12:13) = (PV(10) + PV(9)*Y(12:13))**2

V(14:16) = (PV(12) + PV(11)*Y(14:16))**2

C----------------------------------------------------------------------C

Return

End

C######################################################################C

**3. ADAPT code for the pharmacodynamic analysis of dexamethasone-Glucose**

C**********************************************************************

C	ADAPT	*

C	Version 5	*

C**********************************************************************

C	MODEL - EXAMPLE	*

C	This file contains Fortran subroutines into which the user	*

C	must enter the relevant model equations and constants. *

C	Consult the User's Guide for details concerning the format for	*

C	entered equations and definition of symbols. *

C	1. Symbol- Parameter symbols and model constants	*

C	2. DiffEq- System differential equations	*

C	3. Output- System output equations	*

C	4. Varmod- Error variance model equations	*

C	5. Covmod- Covariate model equations (ITS,MLEM)	*

C	6. Popinit- Population parameter initial values (ITS,MLEM)	*

C	7. Prior - Parameter mean and covariance values (ID,NPD,STS) *

C	8. Sparam- Secondary parameters	*

C	9. Amat - System state matrix	*

C**********************************************************************

C######################################################################C

Subroutine SYMBOL

Implicit None

Include 'globals.inc'

Include 'model.inc'

C----------------------------------------------------------------------C

C	Enter as Indicated	C

C----c-----------------------------------------------------------------C

NDEqs	= 30 !Enter # of Diff. Eqs.

NSParam =4 ! Enter # of System Parameters.

NVparam = 2 ! Enter # of Variance Parameters.

NSecPar = 6	! Enter # of Secondary Parameters.

NSecOut = 0 ! Enter # of Secondary Outputs (not used).i

Ieqsol = 1 ! Model type: 1 - DIFFEQ, 2 - AMAT, 3 - OUTPUT only.

Descr	= 'DEX glucose in horses'

C----------------------------------------------------------------------C

C	Enter Symbol for Each System Parameter (eg. Psym(1)='Kel')	C

C----c-----------------------------------------------------------------C

Psym(1)='kout'

Psym(2)='EC50'

Psym(3)='Emax'

Psym(4)='gama'

C----------------------------------------------------------------------C

C	Enter Symbol for Each Variance Parameter {eg: PVsym(1)='Sigma'}	C

C----c-----------------------------------------------------------------C

PVsym(1)='sigma1'

PVsym(2)='intercept1'

C----------------------------------------------------------------------C

C	Enter Symbol for Each Secondary Parameter {eg: PSsym(1)='CLt'}	C

C----c-----------------------------------------------------------------C

PSsym(1)='kin1'

PSsym(2)='kin2'

PSsym(3)='kin3'

PSsym(4)='kin4'

PSsym(5)='kin5'

PSsym(6)='kin6'

C----------------------------------------------------------------------C

Return

End

C######################################################################C

Subroutine DIFFEQ(T,X,XP)

Implicit None

Include 'globals.inc'

Include 'model.inc'

Real*8 T,X(MaxNDE),XP(MaxNDE)

Real*8 Bmax1,CL10, Kd, Kfs, Kfp, CLr, Fs, Fp

Real*8 Rb_L, fup, Ka1, Kb, Kc,Vj,Fr2,Fr3,CLuf

Real*8 BW, Vp, V1, V2, Qco, Q1, Q2, Rb, MWd, MWs, MWp

Real*8 Kp1_a, Kp1_b, Kp1_c, Kp1_d, Kp1_e, Kp1_f

Real*8 Kp2

Real*8 Kas, Kal, Fml,Kao1,kao2,F1,F2,ktr,kap,kap1,kfp1,Fp1

Real*8 EC50, Emax, gama, kout

Real*8 Kin1,Kin2,Kin3,Kin4,Kin5,Kin6

C----------------------------------------------------------------------C

C	Enter Differential Equations Below {e.g. XP(1) = -P(1)*X(1) }	C

C----c-----------------------------------------------------------------C

kout = P(1)

Emax =P(2)

EC50 = P(3)

gama = P(4)

! Physiological parameters	unit: V-mL, Q-mL/h, 1g =1mL

BW = 550000

Vp = 38896

V1 = 7150

V2 = 503954

Qco=1963540

Q1 = 651550

Q2 =1311990

Rb =0.6945	! 0.664 male 0.725 female

fup = 0.175

MWd=392.46

MWs=492.54

MWp=516.41

Bmax1=140.1

Kp2=1.09

Kd=0.269

CL10=260500

Kfs=20.59

Kfp=16.67

CLr=3131

Fs=0.6951

Fp=1

kas=0.01052

kal=0.1312

kao1=0.5291

kao2=0.4212

Fp1=0.5642

Fml=1

F1=0.6622

F2=0.5210

kap=2.949

Kfp1=30

Kap1=4.126

C....... Model differential equations

Kp1_a= (-(X(2) - Kd -Bmax1) + ((X(2)- Kd

1	-Bmax1)**2+4*Kd*X(2)) **0.5)/(2*Kd)

Kp1_b= (-(X(7) - Kd -Bmax1) + ((X(7)- Kd

1	-Bmax1)**2+4*Kd*X(7)) **0.5)/(2*Kd)

Kp1_c= (-(X(11) - Kd -Bmax1) + ((X(11)- Kd

1	-Bmax1)**2+4*Kd*X(11)) **0.5)/(2*Kd)

Kp1_d= (-(X(15) - Kd -Bmax1) + ((X(15)- Kd

1	-Bmax1)**2+4*Kd*X(15)) **0.5)/(2*Kd)

Kp1_e= (-(X(19) - Kd -Bmax1) + ((X(19)- Kd

1	-Bmax1)**2+4*Kd*X(19)) **0.5)/(2*Kd)

Kp1_f= (-(X(23) - Kd -Bmax1) + ((X(23)- Kd

1	-Bmax1)**2+4*Kd*X(23)) **0.5)/(2*Kd)

c	Dose 1 2005Ref8-IV-DEX DEX-0.05 mg/kg

XP(1) = (Q1*X(2)/Kp1_a

1	+ Q2*X(3)/Kp2 - Qco *X(1)- CLr*X(1))/Vp/Rb

XP(2) = (Q1*(X(1)- X(2)/Kp1_a))/V1-(CL10*X(2)/Kp1_a)/V1

XP(3) = (Q2*(X(1)-X(3)/Kp2))/V2

c	Dose 2 2019Ref13-IM-DEX_ISO-0.03 mg/kg

XP(4) = -Kas*X(4)

XP(5) = Kas*X(4)-Kfs*X(5)

XP(6) = (Kfs*Fs*X(5)*MWd/MWs+Q1*X(7)/Kp1_b

1	+ Q2*X(8)/Kp2 - Qco *X(6)- CLr*X(6))/Vp/Rb

XP(7) = (Q1*(X(6)- X(7)/Kp1_b))/V1-(CL10*X(7)/Kp1_b)/V1

XP(8) = (Q2*(X(6)-X(8)/Kp2))/V2

c	Dose 3	2019Ref13-IV-DEX-PHO low DEX-0.1ug/kg+0.07ug/kg/h 1.556 PM

XP(9) = R(1) - Kfp*X(9)

XP(10) = (Kfp*Fp*X(9)*MWd/MWp+Q1*X(11)/Kp1_c

1	+ Q2*X(12)/Kp2 - Qco *X(10)- CLr*X(10))/Vp/Rb

XP(11) = (Q1*(X(10)- X(11)/Kp1_c))/V1-(CL10*X(11)/Kp1_c)/V1

XP(12) = (Q2*(X(10)-X(12)/Kp2))/V2

c	Dose 4	2019Ref13-IV-DEX-PHO med

XP(13) = R(2) -Kfp*X(13)

XP(14) = (Kfp*Fp*X(13)*MWd/MWp+Q1*X(15)/Kp1_d

1	+ Q2*X(16)/Kp2 - Qco *X(14)- CLr*X(14))/Vp/Rb

XP(15) = (Q1*(X(14)- X(15)/Kp1_d))/V1-(CL10*X(15)/Kp1_d)/V1

XP(16) = (Q2*(X(14)-X(16)/Kp2))/V2

c	Dose 5 2019Ref13-IV-DEX-PHO high

XP(17) = R(3) - Kfp*X(17)

XP(18) = (Kfp*Fp*X(17)*MWd/MWp+Q1*X(19)/Kp1_e

1	+ Q2*X(20)/Kp2 - Qco *X(18)- CLr*X(18))/Vp/Rb

XP(19) = (Q1*(X(18)- X(19)/Kp1_e))/V1-(CL10*X(19)/Kp1_e)/V1

XP(20) = (Q2*(X(18)-X(20)/Kp2))/V2

c	Dose 6	2017Ref14-IM-DEX_ALC DEX-0.05 mg/kg

XP(21) = -Kal*X(21)

XP(22) = (Kal*Fml*X(21)+Q1*X(23)/Kp1_f

1	+ Q2*X(24)/Kp2- Qco *X(22)- CLr*X(22))/Vp/Rb

XP(23) = (Q1*(X(22)- X(23)/Kp1_f))/V1-(CL10*X(23)/Kp1_f)/V1

XP(24) = (Q2*(X(22)-X(24)/Kp2))/V2

C---PD

XP(25) = kin1 * (1+ Emax*X(1)**gama/(EC50**gama+X(1)**gama))

1	- kout*X(25)

Kin1 = IC(25)*kout

XP(26) = kin2 * (1+ Emax*X(6)**gama/(EC50**gama+X(6)**gama))

1	- kout*X(26)

Kin2 = IC(26)*kout

XP(27) = kin3 * (1+ Emax*X(10)**gama/(EC50**gama+X(10)**gama))

1	- kout*X(27)

Kin3 = IC(27)*kout

XP(28) = kin4 * (1+ Emax*X(14)**gama/(EC50**gama+X(14)**gama))

1	- kout*X(28)

Kin4 = IC(28)*kout

XP(29) = kin5 * (1+ Emax*X(18)**gama/(EC50**gama+X(18)**gama))

1	- kout*X(29)

Kin5 = IC(29)*kout

XP(30) = kin6 * (1+ Emax*X(22)**gama/(EC50**gama+X(22)**gama))

1	- kout*X(30)

Kin6 = IC(30)*kout

Return

End

C####################################################################C

Subroutine OUTPUT(Y,T,X)

Implicit None

Include 'globals.inc'

Include 'model.inc'

Real*8 Y(MaxNOE),T,X(MaxNDE)

Integer DelayPar

Real*8 CLr

C----------------------------------------------------------------------C

C	Enter Output Equations Below	{e.g. Y(1) = X(1)/P(2) }	C

C----c-----------------------------------------------------------------C

Y(1) = X(25)

Y(2) = X(26)

Y(3) = X(27)

Y(4) = X(28)

Y(5) = X(29)

Y(6) = X(30)

C----------------------------------------------------------------------C

Return

End

C######################################################################C

Subroutine VARMOD(V,T,X,Y)

Implicit None

Include 'globals.inc'

Include 'model.inc'

Real*8 V(MaxNOE),T,X(MaxNDE),Y(MaxNOE)

C----------------------------------------------------------------------C

C	Enter Variance Model Equations Below	C

C	{e.g. V(1) = (PV(1) + PV(2)*Y(1))**2 }	C

C----c-----------------------------------------------------------------C

V(1:6) = (PV(2) + PV(1)*Y(1:6))**2

C----------------------------------------------------------------------C

Return

End

C######################################################################C
